# High-Efficiency and High-Capacity Aqueous Electrochromic Energy Storage Devices Enabled by Decoupled Titanium Oxide/Viologen Derivative Hybrid Materials

**DOI:** 10.34133/research.0909

**Published:** 2025-10-03

**Authors:** He Zhang, Mingze Sun, Fangyuan Sun, Qing Sun, Ge Cao, Xiaowen Wu, Huan Ling, Fengyu Su, Yanqing Tian, Yan Jun Liu, Lizhi Xu, Yanhong Tian

**Affiliations:** ^1^ State Key Laboratory of Precision Welding & Joining of Materials and Structures, Harbin Institute of Technology, Harbin 150001, China.; ^2^ Department of Mechanical Engineering, The University of Hong Kong, Hong Kong, SAR 999077, China.; ^3^ Advanced Biomedical Instrumentation Centre Limited, Hong Kong, SAR 999077, China.; ^4^ Department of Materials Science and Engineering, Southern University of Science and Technology, Shenzhen 518055, China.; ^5^ Zhengzhou Research Institute, Harbin Institute of Technology, Zhengzhou 450000, China.; ^6^ Department of Electrical and Electronic Engineering, Southern University of Science and Technology, Shenzhen 518055, China.; ^7^ Materials Innovation Institute for Life Sciences and Energy (MILES), The University of Hong Kong Shenzhen Institute of Research and Innovation (HKU-SIRI), Shenzhen 518057, China.

## Abstract

The integration of electrochromism and energy storage within a single platform marks a pioneering approach to multifunctional electronics. However, achieving electrochromic energy storage devices (EESDs) that exhibit both high coloration efficiency and substantial energy capacity remains a formidable challenge, primarily due to the inherent trade-off between these 2 functionalities. Herein, we propose a distinctive strategy for the fabrication of EESDs using a chemically bonded titanium oxide (TiO_2_)/viologen derivative (**TGP**) hybrid material, leveraging their decoupled electrochromic and energy storage mechanisms. The resulting EESDs demonstrate a remarkable coloration efficiency of 512.93 cm^2^/C and an impressive areal capacity of 62.2 mAh/m^2^. By employing molecular engineering, we effectively reduce the bandgap and mitigated radical dimerization of the viologen derivative, resulting in a highly saturated magenta-colored state with exceptional stability. The device retained its performance after 3,000 electrochemical cycles in environmentally benign aqueous electrolytes. Furthermore, integration with a counter Zn anode effectively enhances energy utilization efficiency through an energy retrieval process. This approach paves the way for the development of EESDs using hybrid materials, holding great potential to propel advancements in the field of visual energy storage.

## Introduction

The integration of electrochromic and energy storage devices into a monolithic electrochromic energy storage device (EESD) platform has given rise to a burgeoning field [1–5]. Electrochromic devices, which can dynamically adjust their optical transmittance in response to an applied electric field, are widely applied in various areas, e.g., smart windows [6–10], energy-saving displays [11–16], spectrometers [17], among others. This optical modulation relies on reversible redox reactions in electrochromic materials, a principle that is similar to the operation of electrochemical energy storage device [18–20]. This similarity facilitates the combination of electrochromic device with energy storage device, resulting in EESDs that not only store energy but also provide a visual indication of the remaining energy through changes in their optical properties [21].

In comparison to conventional batteries and supercapacitors, EESDs offer an intuitive means of monitoring energy levels, opening up new possibilities for multifunctional electronics [22–24]. Despite extensive research efforts, achieving high performance in both electrochromism and energy storage remains challenging, primarily due to their inherent trade-off between high coloration efficiency and energy storage capacity [25]. Specifically, high electrochromic efficiency implies low energy consumption for color change, which requires a low charge injection density. In contrast, achieving high energy storage capacity demands high charge densities [26]. This trade-off is especially critical with commonly used electrochromic energy storage materials (e.g., metal oxides). They typically have high energy storage capacity due to their ability to intercalate and deintercalate ions [27,28], while their coloration efficiency is typically limited to ~100 cm^2^/C [29], a consequence of the high injected charge densities. In contrast, electrochromic devices utilizing organic small-molecule electrochromic materials, e.g., viologens (*N*,*N*′-disubstituted-4,4′-bipyridinium derivatives), demonstrate a high coloration efficiency (~500 cm^2^/C) [30,31]. However, their energy storage capacity is typically only a few mAh/m^2^, partly due to their limited ability to store ions [32]. Given these facts, counterbalancing the incompatibility between electrochromism and energy storage in a single material platform is sought after.

Hybrid materials that combine high-capacity metal oxides and highly efficient viologen derivatives may offer an alternative strategy, as they can balance the trade-off between energy storage and electrochromic functions [33,34]. Recently, devices configured with zinc (Zn) counter anodes were exploited to alleviate these conflicts by leveraging their energy recovery capabilities, which can effectively improve energy utilization efficiency [26]. Previous reports demonstrated that integrating these functional hybrids with Zn anodes can further facilitate energy recycling and reduce overall energy consumption [35,36]. However, EESDs still suffer from substantial challenges: (a) Strong bonding is crucial for synchronized electrochemical reactions, enabling the simultaneous functionality of the individual components in the hybrid materials [34]. (b) The use of aqueous electrolytes in Zn anode electrochromic devices can lead to the dimerization of radicals, resulting in poor optical modulation stability (fewer than 1,000 cycles). This requires careful design of the molecular structure [37,38]. (c) Electrochromic function and energy storage in hybrid materials tend to decouple, necessitating a careful clarification of the working principles [1].

In this work, we propose a unique decoupling strategy that allocates the electrochromic and energy storage functions to different components within a hybrid system, thereby balancing the trade-off between coloration efficiency and energy storage capacity. Our previous work shows that viologen derivatives with phosphate groups can chemically combine with TiO_2_ nanoporous films through P–O–Ti bonds, which provides a feasible method for the integration of metal oxides and viologen derivatives [39]. The robustly integrated TiO₂–viologen hybrid system, in which TiO₂ primarily contributes to energy storage while viologen is mainly responsible for electrochromism, simultaneously achieves high electrochromic efficiency and large energy storage capacity in aqueous Zn-based EESDs. The reliable integration of the newly designed viologen derivative **TGP** and TiO_2_ film enables the synchronous electrochemical process. The strategic introduction of thiophene groups into the chromophore effectively narrows the bandgap and suppresses radical dimerization, thereby enabling a highly saturable magenta-colored state and effectively extending the operational lifetime of viologen-based electrochromic devices in aqueous environments. Moreover, this work successfully realizes a vividly magenta-colored EESD, thereby expanding the limited color palette of conventional EESDs beyond metal oxides and conducting polymers. These findings may provide valuable insights into the design of hybrid electrochromic energy storage materials with decoupled working principles, thereby offering new possibilities for advanced energy supply platforms, display electronics, smart windows, and beyond.

## Results

**Fig. 1.**
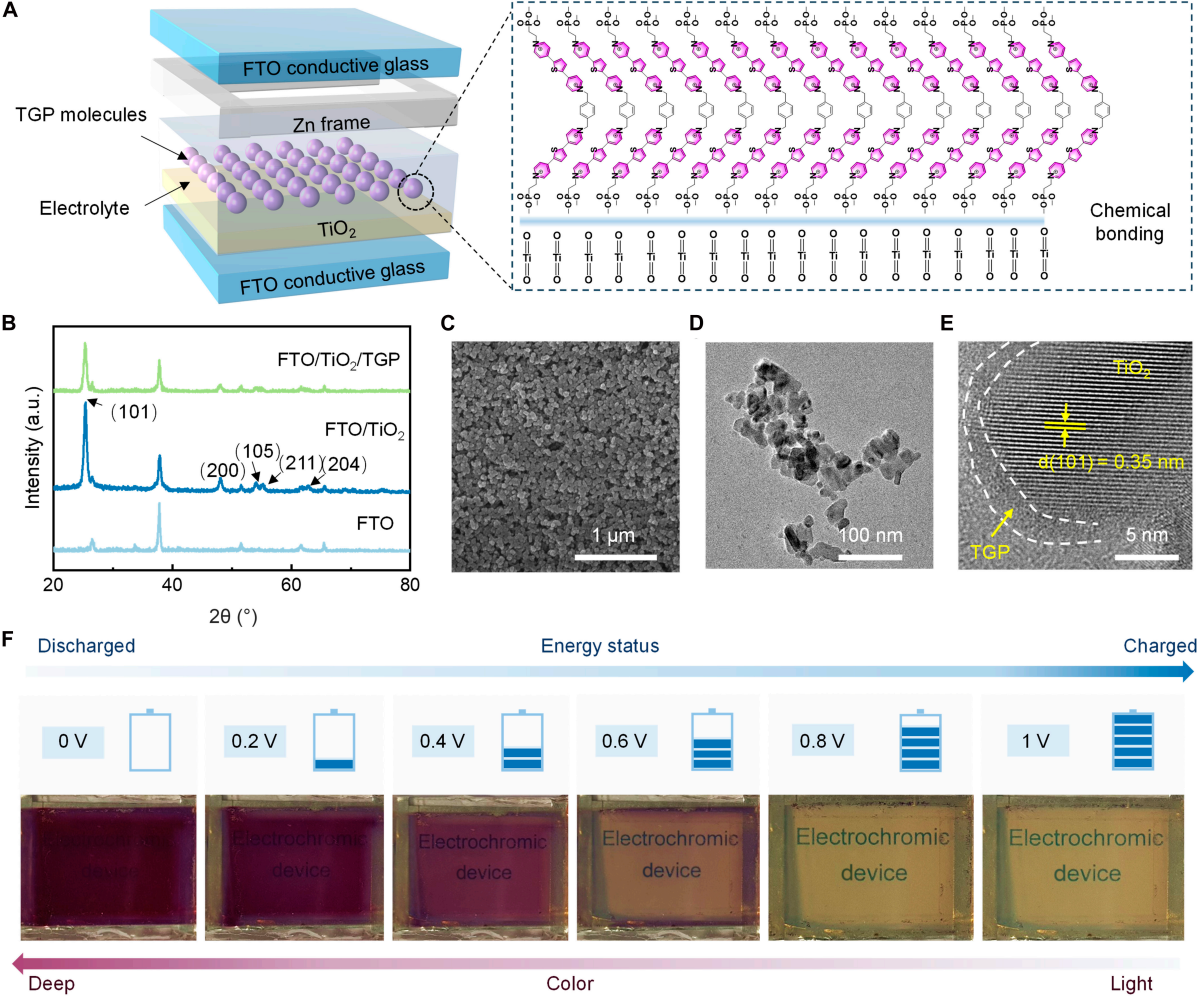
Structural and material characteristics of TiO_2_/TGP electrochromic energy storage devices (TT-EESDs). (A) Schematics of the configuration of the TT-EESDs, including TiO_2_/TGP hybrid materials, a Zn anode with frame structure as counter electrode, and a Zn^2+^ aqueous solution as electrolyte. (B) XRD patterns of TiO_2_ and TiO_2_/TGP film on FTO conductive glass. (C) SEM image of TiO_2_/TGP film. (D and E) TEM (D) and HR-TEM (E) of TiO_2_/TGP nanoparticles. (F) Optical images of TT-EESDs at different energy statuses.

The TiO_2_/**TGP**-EESDs (TT-EESDs) are structured in a 6-layer configuration. The substrate is fluorine-doped tin oxide (FTO) conductive glass, onto which a porous TiO_2_ film is deposited. This is followed by **TGP** molecules, an aqueous electrolyte containing Zn^2+^ ions, a Zn frame, and a transparent top glass layer (Fig. 1A). A uniform TiO_2_ nanoparticle film is fabricated by blade-coating TiO_2_ paste onto FTO glass, followed by thermal annealing. The **TGP**, a newly designed viologen-based molecule, is then incorporated into the aqueous medium. The robust attachment of **TGP** molecules to TiO_2_ is facilitated through strong P–O–Ti chemical bonds between the phosphate groups of **TGP** and the oxygen vacancies on the TiO_2_ surface [33], as confirmed by Fourier transform infrared (FTIR) spectroscopy (Fig. S10). This strong chemical interaction is crucial for ensuring synchronous electrochemical processes, allowing the TiO_2_ and **TGP** to function in a coordinated manner.

X-ray diffraction (XRD) (Fig. 1B) and scanning electron microscopy (SEM) (Fig. 1C) reveal that the TiO_2_ nanoparticles undergo sintering, resulting in a porous film with an anatase crystal structure, which provides a large surface area for Zn^2+^ storage. Further characterization using transmission electron microscopy (TEM) (Fig. 1D), high-resolution TEM (HRTEM) (Fig. 1E), energy dispersive spectroscopy (EDS) (Fig. S11), and optical imaging (Fig. S12) demonstrates that **TGP** molecules are anchored onto the TiO_2_ nanoparticles, forming an amorphous **TGP** layer that encapsulates the crystalline TiO_2_, thereby supporting the synchronized electrochemical process. Subsequently, a Zn frame is positioned along the perimeter of the film, and a Zn-ion electrolyte [e.g., Zn(OTF)_2_] is applied. The entire assembly is then covered with a transparent glass layer. Figure 1F illustrates the device’s ability to modulate color in response to varying voltages, visually representing the energy storage and release processes.

**Fig. 2.**
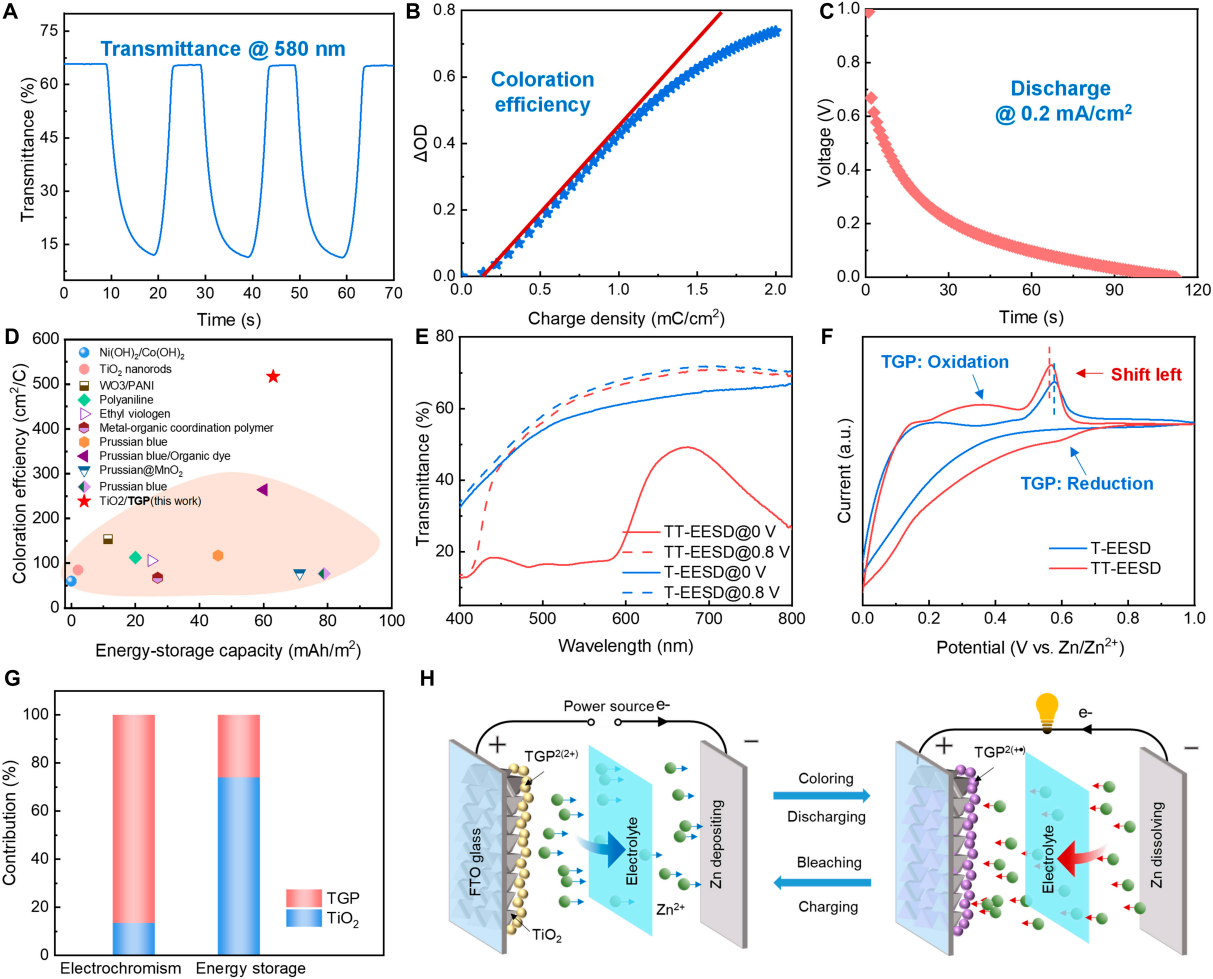
Decoupled working principle of TiO_2_ and TGP in TT-EESDs. (A) Colored/bleached transmission curves at a wavelength of 580 nm of the TT-EESDs with applying voltages of 0/1 V, which show a switch time of 4.0/3.6 s. (B) Coloration efficiency of the TT-EESDs. (C) Discharge curves at a current density of 0.2 mA/cm^2^. (D) Comparison of coloration efficiency and energy storage capacity of TT-EESDs with previously reported devices based on metal oxides, polymers, and Prussian blue, which are famous for their bifunctional property. (E) Comparison of transmittance spectrum in a wavelength range from 400 to 800 nm of T-EESDs and TT-EESDs. (F) Comparison of cyclic voltammetry (CV) curves of T-EESDs and TT-EESD at the same scan rate. (G) Decoupled contributions of TiO_2_ and TGP for electrochromism and energy storage in TT-EESDs. (H) Schematic illustration of the working principle of TT-EESDs.

We investigated the electrochromic and energy storage performance of the Zn-anode TT-EESD. The visible absorbance of TT-EESDs in a wavelength range from 400 to 800 nm is highly dependent on the applied external electric field, with a pronounced coloration process occurring as the voltage is increased (Fig. S14). Within a sweeping voltage window of 0 to 1 V, the transmittance was periodically regulated between 66% and 12% at a wavelength of 580 nm, indicating an optical contrast of approximately 54% (Fig. 2A). Notably, the TT-EESD demonstrated a coloration efficiency of 512.93 cm^2^/C (Fig. 2B) and a high areal capacity of 62.2 mAh/m^2^ at 0.2 mA/cm^2^ (Fig. 2C). Impressively, in comparison to state-of-the-art EESDs based on materials like metal oxides [40,41], polymers [42–44], and Prussian blue [45–48], small molecules [32] and TT-EESDs demonstrate superior comprehensive performance in both coloration efficiency and energy storage capacity (Fig. 2D and Table S1). These findings confirmed that the hybrid TiO_2_ and **TGP** materials effectively mitigated the trade-off.

To investigate the improvement mechanism, we compared the electrochromic and energy storage performance with the pure TiO₂-based EESDs (T-EESDs). The T-EESDs exhibited negligible optical modulation capability across the 400- to 800-nm-wavelength range when different voltages were applied (Fig. 2E). In contrast, a distinct transmittance difference was observed between the colored and bleached states in the TT-EESDs, indicating that the electrochromic properties are primarily governed by the **TGP** molecules. Additionally, cyclic voltammetry (CV) curves evaluated the electrochemical and energy storage properties of both devices (Fig. 2F). The CV curve of the TT-EESDs presents a pair of different redox peaks, corresponding to the reversible transition between the colorless dicationic state [**TGP**^2(2+)^] and the magenta-colored radical cation state [**TGP**^2(+•)^]. The simultaneous appearance of characteristic peaks from both TiO_2_ and **TGP** in the TT-EESD curve suggests a stable interaction between the 2 materials, which facilitates synchronized electrochemical processes. Notably, a leftward shift of the oxidation peak of TiO_2_ in TT-EESDs indicates an enhancement in the deintercalation of Zn^2+^ ions from TiO_2_. This shift may be attributed to the increased electron cloud density in TiO_2_, resulting from the formation of covalent bonds between TiO_2_ and **TGP** [49,50]. It is noteworthy that the energy storage capacity is closely correlated with the area enclosed by the CV curves. The modest increase in the CV curve area after the incorporation of **TGP** molecules demonstrates that TiO_2_ is primarily responsible for energy storage, as it facilitates the insertion and extraction of Zn^2+^ ions from the TiO_2_ lattice.

Furthermore, the quantitative contributions of **TGP** and TiO_2_ to electrochromism and energy storage (Fig. 2G) were decoupled based on the transmittance difference at 580 nm (Fig. 2E) and the area of the CV curves (Fig. 2F). TiO_2_ contributes only 13.5% to electrochromism, but accounts for 74% of the energy storage capacity. In contrast, **TGP** plays a dominant role in optical modulation, contributing 86.5% to electrochromism, but only 26% to energy storage. These results underscore that the hybrid TiO_2_/**TGP** material system achieves synchronized electrochromism and energy storage performance through decoupled mechanisms.

Finally, the operational principle of this bifunctional platform is depicted schematically in Fig. 2H (see details in Note S2). During the reversible electrochemical cycling of the TiO_2_/**TGP** electrode, **TGP** molecules transition between the **TGP**^2(+•)^ (colored state) and **TGP**^2(2+)^ (bleached state) for electrochromism (Fig. S16). Simultaneously, the intercalation and deintercalation of the Zn^2+^ dominate the energy storage process at the porous TiO_2_, exhibiting fast kinetics (Fig. S17), high capacity, and high reversibility. Moreover, the countered Zn/Zn^2+^ redox pair provides the device with energy recovery capabilities, further enhancing its overall energy efficiency.

**Fig. 3.**
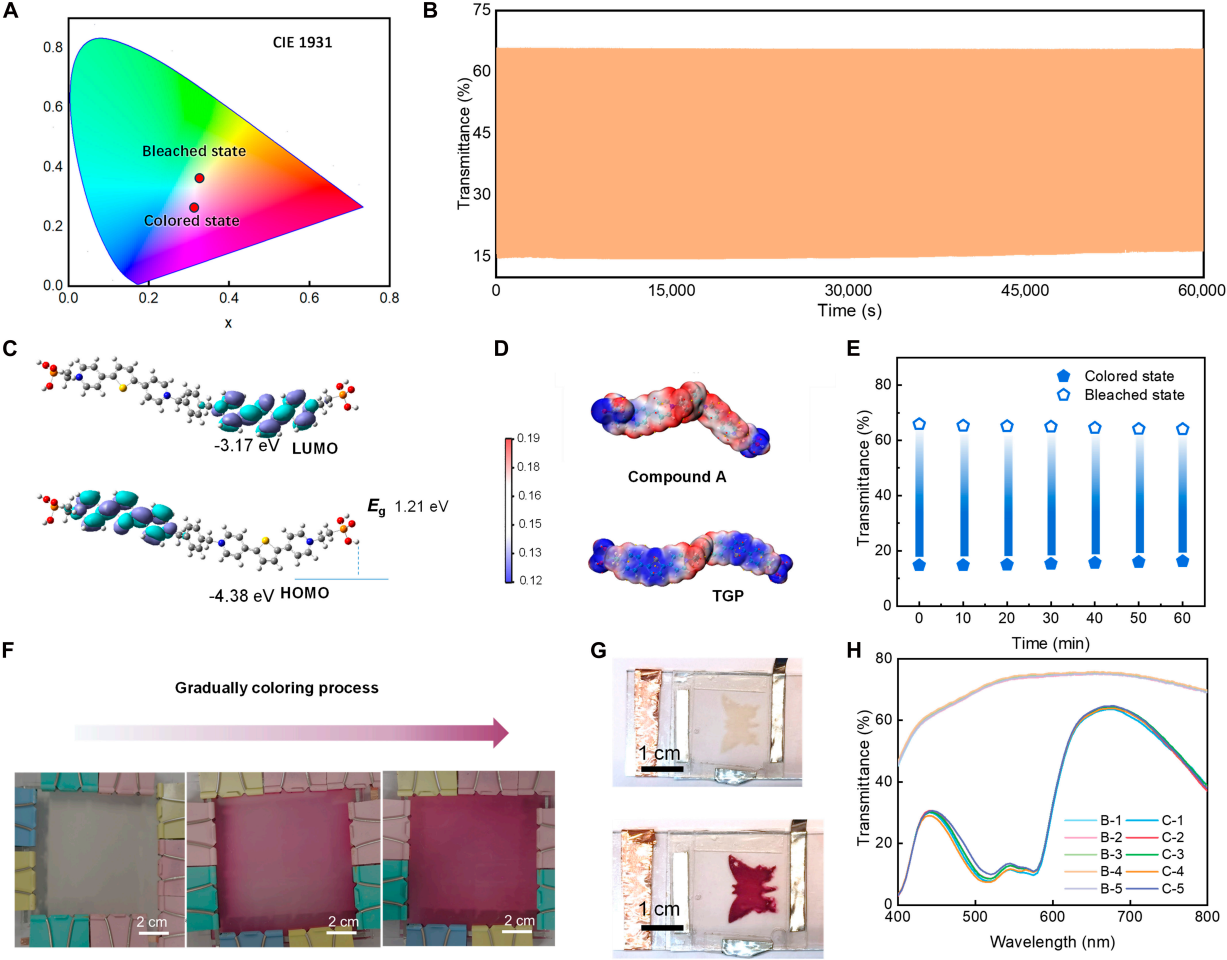
Electrochromic properties of TT-EESDs. (A) Peak*x*y* color space 481 (CIE 1931) results of TT-EESDs at the colored and bleached state. (B) Cyclic stability of transmittance in a voltage window 0 V/1 V within 3,000 cycles. (C) Schematics of HOMO and LUMO distribution of TGP at the dication state. (D) Electrostatic potential distribution of Compound A and TGP. (E) Bistability after applying a bias of 0 V/1 V for 30 s. (F) A prototype of large-area TT-EESD (10 cm × 10 cm). The scale bar is 5 cm. (G) A prototype of colorful display with butterfly patterned TT-EESD. (H) The transmittance curves of the large-area TT-EESD (10 cm × 10 cm) at 5 different regions.

The TT-EESD exhibits a distinctive and vibrant magenta-colored state, a unique feature of the device. Through colorimetric characterization, the color transition between the bleached and colored states has been quantified (Fig. 3A and Fig. S18). In the CIE 1931 color space, the parameters L*, a*, and b* correspond to lightness, redness/greenness, and yellowness/blueness, respectively. In the fully colorless state, the coordinates (L*, a*, b*) are (72.65, −2.43, 6.45), transitioning to (46.51, 19.79, −14.46) in the fully colored state. The obvious increase in the a* value from −2.43 to 19.79 highlights a marked enhancement in redness, consistent with a shift toward the magenta region (Table S2). This vibrant magenta-colored state is attributed to the chromophore’s acceptor–donor–acceptor (A–D–A) configuration, achieved by introducing thiophene units between 2 pyridine moieties. This modification effectively reduces the bandgap of the π–π* transition, resulting in a blue shift of the absorption band and the emergence of the vivid magenta hue [51].

The TT-EESD demonstrates exceptional optical contrast, maintaining stability after 3,000 coloring–bleaching cycles over a voltage range of 0 to 1 V (Fig. 3B and Fig. S19), outperforming previously reported viologen-based electrochromic devices in aqueous electrolytes [37]. This remarkable stability was realized through the unique molecular engineering, achieved by the incorporation of a thiophene group between dipyridyl moieties. Compared with the **Compound A** reported in the previous report [39], the energy gap (*E*_g_) of **TGP** is smaller (Fig. 3C, Fig. S20, and Table S3), which is beneficial for redox reversibility and stability [52]. The electrostatic potential (ESP) was employed to assess the dimerization propensity of **TGP**^2(+•)^ and **Compound A**^2(+•)^ molecules (Fig. 3D). In the **TGP** molecule, lower ESP values near the thiophene moiety (the primary site of initial contact) result in reduced interactions between the terminal rings, mitigating specific dimerization reactions and thereby enhancing device performance.

Additionally, the TT-EESD exhibits an outstanding memory effect, capable of preserving either the colored or bleached state for over 60 min after a brief 30-s application of a 0 V/1 V bias (Fig. 3E). This bistable behavior represents a remarkable advancement over viologen-based devices and is attributed to the robust anchoring of **TGP** onto the TiO_2_ substrate, which ensures stability by preventing spontaneous discharge. Large-area (10 cm × 10 cm) (Fig. 3F) and patterned (Fig. 3G and Fig. S21) TT-EESDs have been successfully fabricated, exhibiting uniform color transitions and excellent optical contrast. These devices demonstrate compatibility with various applications, such as smart portholes and energy-efficient displays. The consistent transmittance curves (Fig. 3H) and CIE coordinates (Figs. S22 and S23 and Table S4) across different regions confirm the uniformity of TT-EESDs, even for areas as large as 100 cm^2^. Notably, the self-coloring process for large-area and patterned devices is achieved by short-circuiting the Zn and TiO_2_/**TGP** electrodes. This unique feature of the Zn-anode TT-EESD facilitates spontaneous electrochemical reactions for self-coloring (Fig. S24). Furthermore, quantitative analysis indicates that the energy consumption of 11.5 mWh/cm^2^ during each coloring cycle can be fully recovered, thereby enhancing the overall energy efficiency (Fig. S25).

**Fig. 4.**
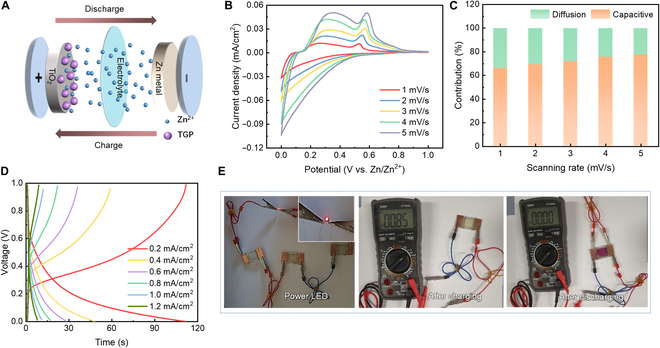
Energy storage performances of TT-EESDs. (A) Schematic illustration of the energy storage process. (B) CV curves at different scan rates (1, 2, 3, 4, and 5 mV/s). (C) Contribution ratio of capacitive at various scan rates (1, 2, 3, 4, and 5 mV/s). (D) Galvanostatic charge–discharge curves at different current densities (0.2, 0.4, 0.6, 0.8, 1.0, and 1.2 mA/cm^2^). (E) Photographs showing an LED powered by the TT-EESDs, after fully charging and discharging.

The TT-EESD exhibits not only exceptional electrochromic performance but also remarkable energy storage capabilities. Structurally, the TT-EESD functions as an aqueous Zn-ion energy storage device, where metallic Zn acts as the anode and the TiO_2_/**TGP** layer, capable of intercalating Zn^2+^ ions, serves as the cathode. Mobile Zn^2+^ ions in the electrolyte facilitate ion transport (Fig. 4A). The CV curves recorded at varying scan rates (1 to 5 mV/s) demonstrate an increasing enclosed area with higher scan rates, indicating the device’s excellent rate capability (Fig. 4B). Utilizing the peak currents at different scan rates and the Randles–Sevcik equation, the diffusion coefficient was calculated to be 9.58 × 10^−9^ cm^2^/s (Fig. S26A) [53]. This value surpasses that of monovalent Li^+^, which can be attributed to the porous anatase TiO_2_ structure, sintered from nanoparticles, and the low-viscosity aqueous environment. Additionally, the slope *b* of log *i* versus log *v* was determined to be 0.76, suggesting a synergistic process involving both pseudocapacitive and diffusion-controlled behaviors (Fig. S26B) [54,55].

To further elucidate the nature of the charge storage, the ratio of capacitive to diffusion contributions was quantitatively analyzed. The capacitive contribution portion, represented by the red region in Fig. S27, increases from 66.05% to 77.63% as the scan rate increases from 1 to 5 mV/s, indicating a capacitance-dominated process (Fig. 4C and Table S5). The rapid Zn^2+^ charge/discharge kinetics also contribute to a high-rate capability. This behavior was validated by examining the discharge time and capacity as functions of discharge current density (Fig. 4D and Fig. S29). At a current density of 0.2 mA/cm^2^, the discharge time and capacity reached 112 s and 62.2 mAh/m^2^, respectively. However, at a higher current density of 1.2 mA/cm^2^, these values decreased to 8 s and 26.7 mAh/m^2^.

The Ragone plot (Fig. S30) highlights the TT-EESD’s impressive energy density of 31.1 mWh/m^2^ at a power density of 1,000 mW/m^2^, demonstrating its superior energy storage performance. The LED powered by the TT-EESD remained illuminated for up to 3.5 h, although its brightness gradually diminished as the device discharged (Fig. 4E and Fig. S31). Notably, the device transitions to a bleached state upon charging, maintaining an output voltage of 0.85 V. Conversely, rapid discharge through electrode short-circuiting induces a vibrant magenta-colored state, further affirming the TT-EESD’s visually interpretable energy storage functionality.

**Fig. 5.**
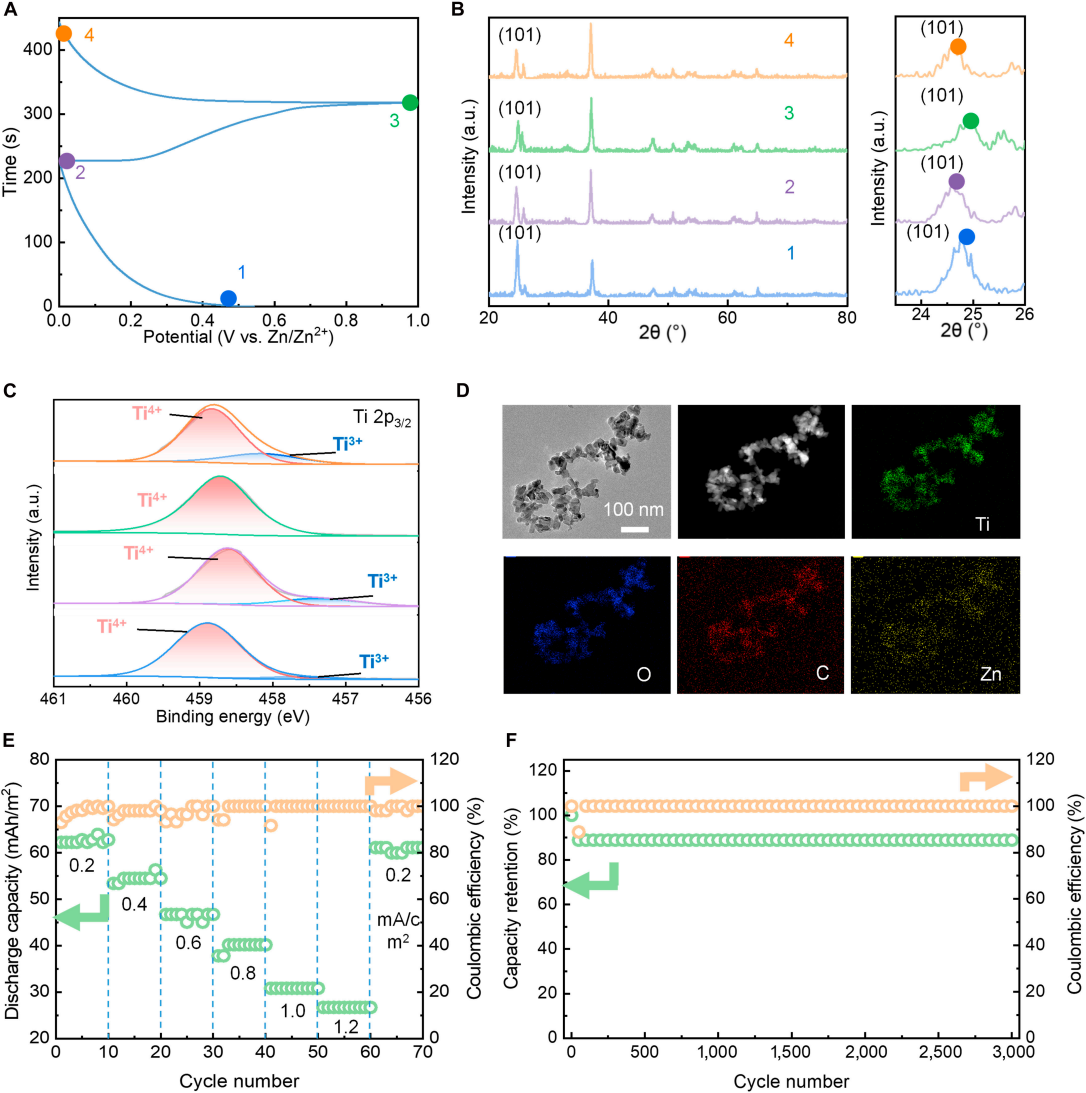
Ex situ characterization of TT-EESDs. (A) Charge–discharge curves at a current density of 0.2 mA/cm^2^. (B) XRD patterns and (C) XPS spectra corresponding to different states (1 to 4) in Fig. 4A. (D) Elemental mapping after inserting Zn^2+^. (E) Cyclic stability and coulombic efficiency at different current densities. (F) Cyclic stability and coulombic efficiency within 3,000 cycles at a current density of 5 mA/cm^2^.

To elucidate the Zn^2+^ storage mechanism in TiO_2_, ex situ XRD and x-ray photoelectron spectroscopy (XPS) were performed corresponding to the states depicted in Fig. 5A. Specifically, peak shifts under different states (1 to 4) were analyzed, with the (101) crystal plane serving as a representative example. During discharge (states 1 and 2 and states 3 and 4), a slight leftward shift was observed, indicating an increase in d-spacing. Conversely, the charging process (states 2 and 3) resulted in a gradual rightward shift, reflecting a decrease in d-spacing. These reversible shifts suggest excellent Zn^2+^ intercalation/deintercalation behavior (Fig. 5B and Fig. S17) [56]. The XPS spectra of Ti 2p_3/2_ further support this mechanism. A peak attributed to Ti^3+^ emerged in the discharged states (states 2 and 4), signifying the reduction of Ti^4+^ in TiO_2_ to Ti^3+^ in Zn*_x_*TiO_2_ due to Zn^2+^ intercalation (Fig. 5C). EDS mappings revealed overlapping regions for Zn and Ti elements in the discharged state, providing additional confirmation of Zn ion insertion into TiO_2_ (Fig. 5D).

The TT-EESD’s rate capability was evaluated at varying charge/discharge current densities. At a high current density of 1.2 mA/cm^2^, a reversible capacity of 26.7 mWh/cm^2^ was achieved. Moreover, when the current density was reduced to 0.2 mA/cm^2^, the capacity returned to its initial value with negligible loss, demonstrating excellent reversibility (Fig. 5E). The device also exhibited remarkable stability, retaining performance after 3,000 charge/discharge cycles at a high current density of 5 mA/cm^2^, underscoring its suitability for practical applications (Fig. 5F and Fig. S32). The capacity exhibits a slight decline during the first 50 cycles before stabilizing, which may be attributed to the formation process of the SEI layer [57]. This outstanding stability is likely due to the conformal encapsulation of **TGP** molecules, which effectively mitigates Zn^2+^ insertion-induced volume expansion. This molecular design ensures the structural integrity of the TiO_2_ framework, enabling sustained performance over prolonged cycling.

## Discussion

In summary, we present a novel strategy for fabricating a visual energy storage platform that effectively integrates both electrochromic and energy storage functions. The TiO_2_/**TGP** hybrid material successfully achieves high coloration efficiency and energy storage capacity, overcoming the intrinsic trade-off between these 2 properties. The decoupled mechanisms governing its electrochromic and energy storage performances offer a promising approach for the design of high-performance EESDs. Notably, molecular structural modifications play a pivotal role in attaining both exceptional cyclic stability in aqueous electrolytes and the vibrant magenta-colored state. This approach expands the design toolkit for creating electrochromic molecules with tunable properties. Furthermore, the TiO_2_/**TGP** material is compatible with an environmentally friendly and safe Zn-anode system, making our TT-EESDs advantageous for reducing energy consumption via energy retrieval processes. Future exploration of bifunctional electrochromic and energy storage materials, coupled with advancements in device structures and underlying mechanisms, holds great potential for driving further innovations in multifunctional electronics.

## Methods

Information about methods used for this research is available in the Supplementary Materials.

## Data Availability

All data needed to evaluate the conclusions in the paper are present in the paper and/or the Supplementary Materials.

## Supplementary Materials

Supplementary 1 Notes S1 and S2 Figs. S1 to S32 Tables S1 to S6
