# Supplementary material for: High-Efficiency and High-Capacity Aqueous Electrochromic Energy Storage Devices Enabled by Decoupled Titanium Oxide/Viologen Derivative Hybrid Materials
Source: Research (Wash D C). 2025 Oct 3;8:0909. doi: 10.34133/research.0909 (PMC12491783; doi:10.34133/research.0909)
Supplement: Supplementary 1 — Notes S1 and S2 Figs. S1 to S32 Tables S1 to S6 [file research.0909.f1.docx]

Supporting Information

High-Efficiency and High-Capacity Aqueous Electrochromic Energy Storage Devices Enabled by Decoupled Titanium Oxide/Viologen Derivative Hybrid Materials

*He Zhang, Mingze Sun, Fangyuan Sun, Qing Sun, Ge Cao, Xiaowen Wu, Huan Ling, Fengyu Su, Yanqing Tian, Yan Jun Liu, Lizhi Xu, Yanhong Tian*

***Supplied Note 1***

**Experimental**

**Synthesis of TGP**

The synthetic route of **TGP** was given in **Figure S1**. In a 100 mL degassed two-necked flask, **Compound 2** (1 g, 4.9 mmol), tetrakis(triphenylphosphine) palladium(0) (204 mg, 0.176 mmol), and potassium phosphate (2.98 g, 14.1 mmol) were combined. Following this, 1,4-dioxane (70 mL) and 2,5-dibromothiophene (**Compound 1**, 424 mg, 1.77 mmol) were introduced into the mixture. The resulting suspension was stirred continuously at 90 °C for 72 hours. After cooling to room temperature, the precipitate was isolated by filtration and washed with CHCl_3_. Concentration of the filtrate yielded a dark brown residue. This residue was subjected to further purification via fresh silica gel column chromatography, using a CHCl_3_ and MeOH mixture (200:1 in volume) as eluent, affording **Compound 3** (2, 5-di(pyridin-4-yl) thiophene) as a light yellow solid (254 mg, 1.06 mmol) with a 60% yield. ^1^H NMR (400 MHz, DMSO-d_6_) δ 8.68 - 8.53 (m, 4H), 7.91 (s, 2H), 7.75-7.60 (m, 4H). (**Figure S2**)

Then, **Compound 3** (400 mg, 1.68 mmol) and diethyl bromoethyl phosphonate (**Compound 4**, 410 mg, 1.68 mmol) were combined in 10 mL of N,N-dimethylformamide (DMF) and heated with stirring at 90 °C for 24 hours. Upon cooling to room temperature, 10 mL of water was added to the solution. The resulting mixture was extracted with ethyl acetate (3 × 20 mL). The combined organic phases were then washed with water (3 × 20 mL) and 30 mL of saturated NaCl solution. Afterward, the organic phase was evaporated under vacuum using a rotary evaporator. The crude product obtained was subsequently purified by alumina column chromatography, employing a chloroform and methanol mixture (15:1 in volume) as eluent. This purification process afforded [1-(2-(diethoxyphosphoryl) ethyl)-4-(5-(pyridin-4-yl) thiophen-2-yl) pyridin-1-ium] (**Compound 5**) as a light yellow solid (350 mg, 0.726 mmol) with a yield of 43%. ^1^H NMR (400 MHz, Deuterium Oxide) δ 8.71-8.52 (m, 2H), 8.37 (d, J=5.5 Hz, 2H), 8.09-7.98 (m, 2H), 7.59 -7.49 (m, 2H), 4.15-3.90 (m, 4H), 2.62 (dt, J=18.3, 7.0 Hz, 2H), 1.16 (t, J=7.1 Hz, 6H). (**Figure S3**)

A solution containing **Compound 5** (341.5 mg, 0.71 mmol) and 1,4-bis(bromomethyl)benzene (**Compound 6**, 91 mg, 0.35 mmol) in 5 ml of DMF was heated at 90°C with stirring for 24 hours. Afterward, the precipitate was filtered off. The filtrate was reheated, and the process was repeated until no further solid formed. The resulting precipitate was filtered and washed with hot DMF, then dried under vacuum to yield a yellow solid (250 mg, 0.204 mmol) identified as 4-(5-(1-(2-(diethoxyphosphoryl)ethyl)pyridin-1-ium-4-yl)-thiophen-2-yl)-1-(4-((4-(5-(1-(2-(diethoxyphosphoryl) ethyl) pyridin-1-ium-4-yl)thiophen-2-yl)pyridin-1-ium-1-yl)methyl)benzyl)pyridin-1-ium-2-ide tetra-bromide, namely **Compound 7**. ^1^H NMR (400 MHz, D_2_O): δ (ppm) 8.94 (d, J = 5.6 Hz, 8H), 8.41 (dd, J = 15.8, 6.6 Hz, 8H), 8.21 (dd, J = 9.4, 4.0 Hz, 4H), 7.68 (d, J = 2.8 Hz, 4H), 5.90 (s, 4H), 4.97 (dd, J = 12.1, 4.6 Hz, 8H), 4.40-4.08 (m, 8H), 2.96-2.70 (m, 4H), 1.31 (t, J = 7.0 Hz, 12H). ^13^C NMR (101 MHz, D_2_O): δ (ppm) 148.66, 148.41, 144.87, 144.63, 142.97, 142.69, 134.53, 133.30, 133.20, 130.06, 123.82, 123.59, 63.99, 63.93, 63.28, 54.97, 54.92, 26.64, 25.23. HRMS m/z calcd for C_48_H_56_O_6_N_4_P_2_S^4+^ ([M]^4+^) 227.57736, found 227.57785. (**Figures S4** and **S5**)

200 mg (0.163 mmol) of **Compound 7** was introduced into a solution of 20 wt% hydrochloric acid (40 mL). The resulting mixture underwent heating at 100 °C for 24 hours with continuous stirring. Subsequently, the solvent was removed under vacuum, and the residue was treated with ethanol to induce precipitation of the desired compound. The precipitated compound was then filtered and dried under vacuum, yielding a pale yellow solid (140 mg, 0.126 mmol) as 4-(5-(1-(2-phosphonoethyl)pyridin-1-ium-4-yl)thiophen-2-yl)-1-(4-((4-(5-(1-(2-phosphonoethyl)pyridin-1-ium-4-yl)thiophen-2-yl)pyridin-1-ium-1-yl)methyl)benzyl)pyridin-1-ium-2-idetetra-bromide, namely **TGP**, with a yield of 77%. ^1^H NMR (400 MHz, Deuterium Oxide) δ 8.76 (dd, *J* = 7.1, 2.2 Hz, 8H), 8.30–8.11 (m, 8H), 8.03 (s, 4H), 7.48 (s, 4H), 5.72 (s, 4H), 4.67–4.63 (m, 4H), 2.39–2.08 (m, 4H). (**Figures S6**-**S8** )

**
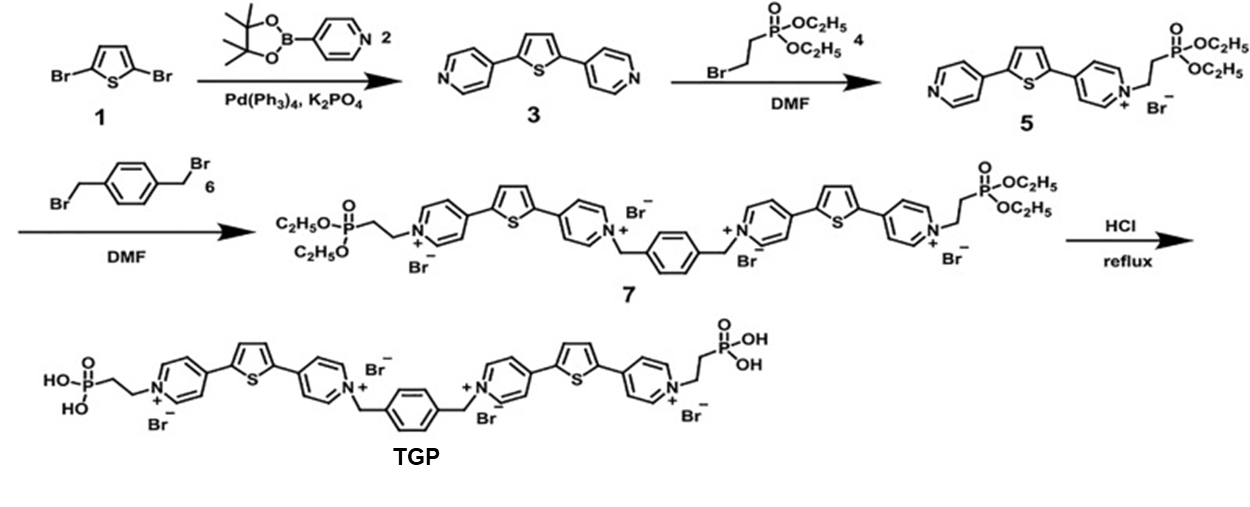
**

**Figure S1 Synthesis of TGP.** The synthetic route of **TGP**.

**
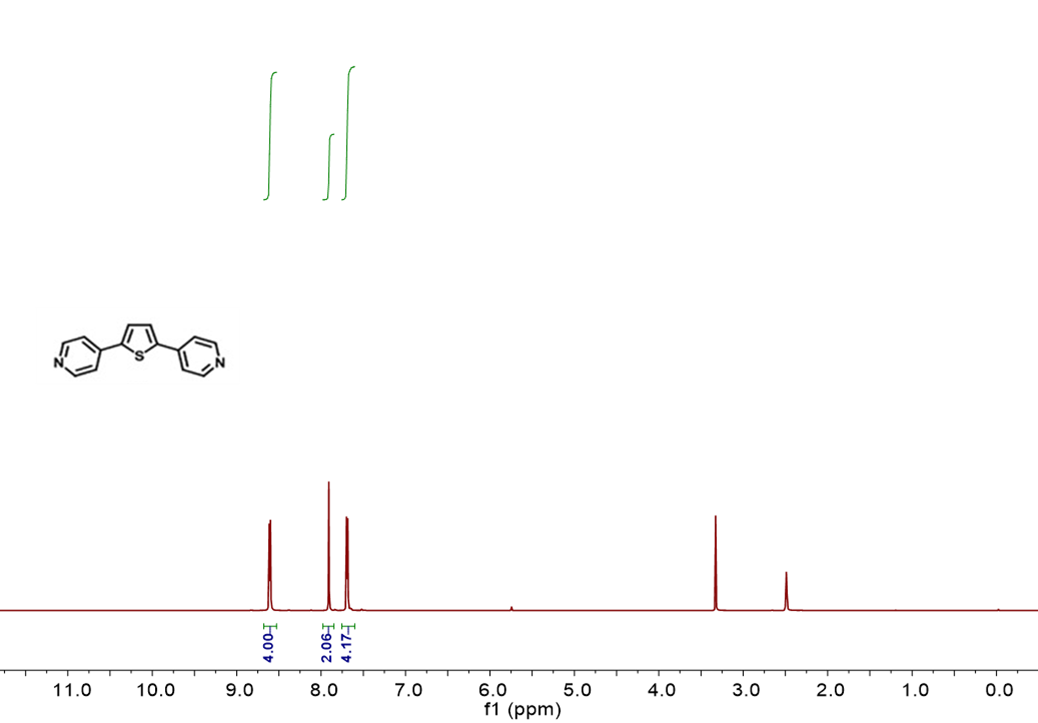
**

**Figure S2** **Characterization of the Compound 3.**  ^1^H NMR of **Compound 3**

**
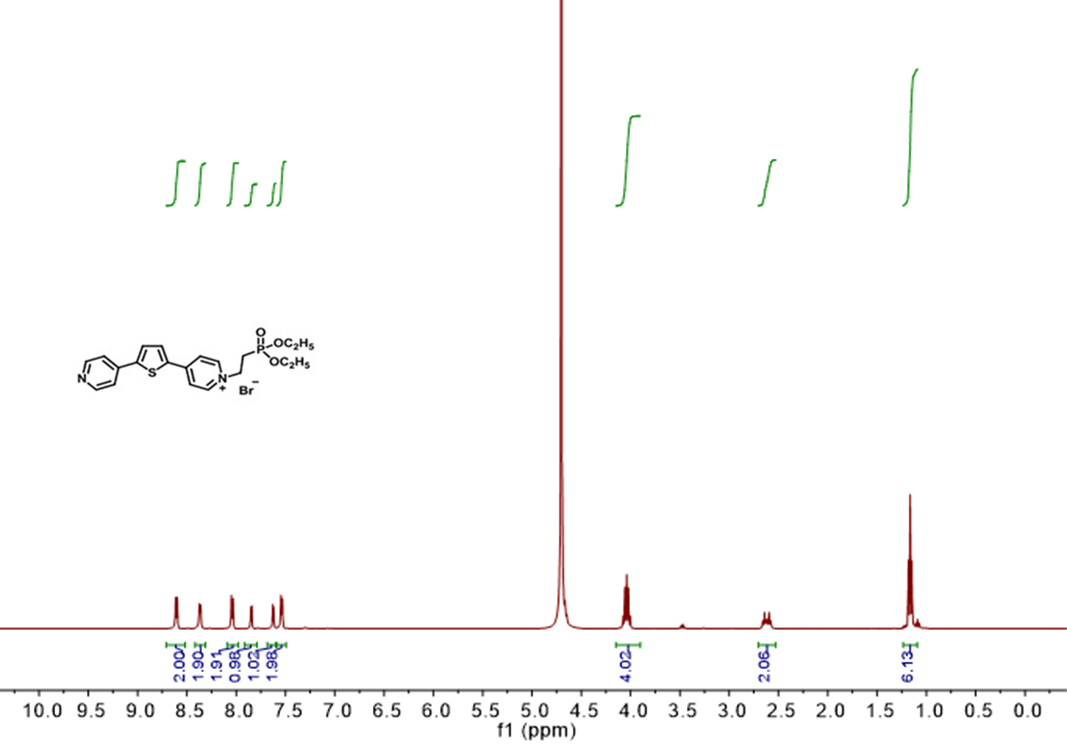
**

**Figure S3** **Characterization of the Compound 5.**  ^1^H NMR of **Compound 5**


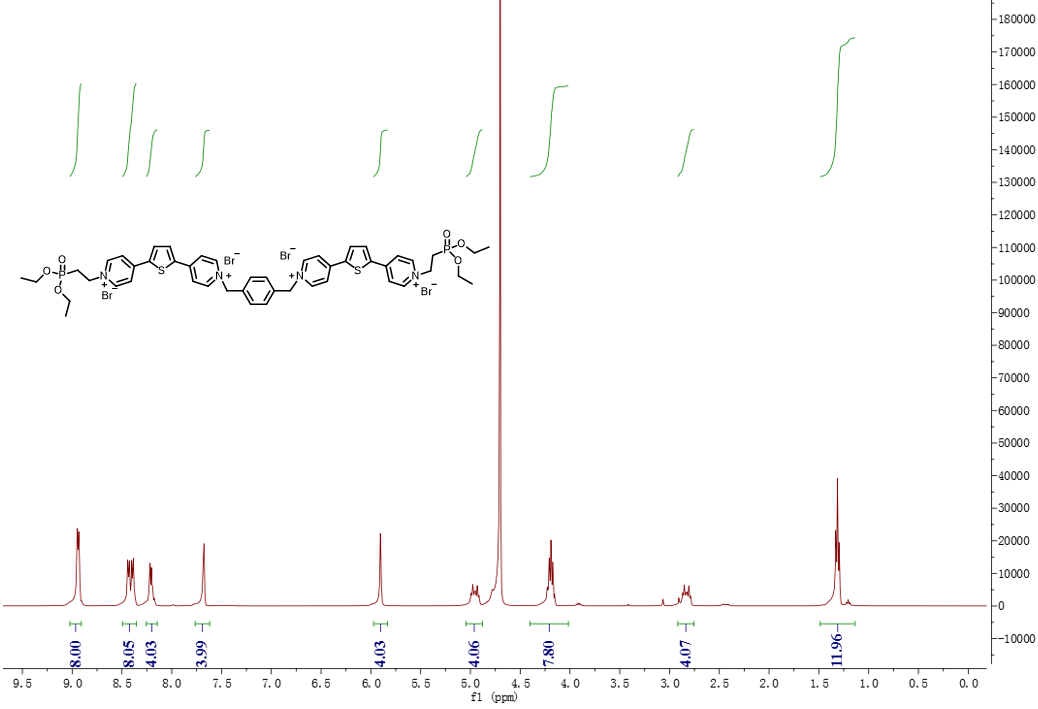


**Figure S4** **Characterization of the Compound 7.**  ^1^H NMR of **Compound 7**

**
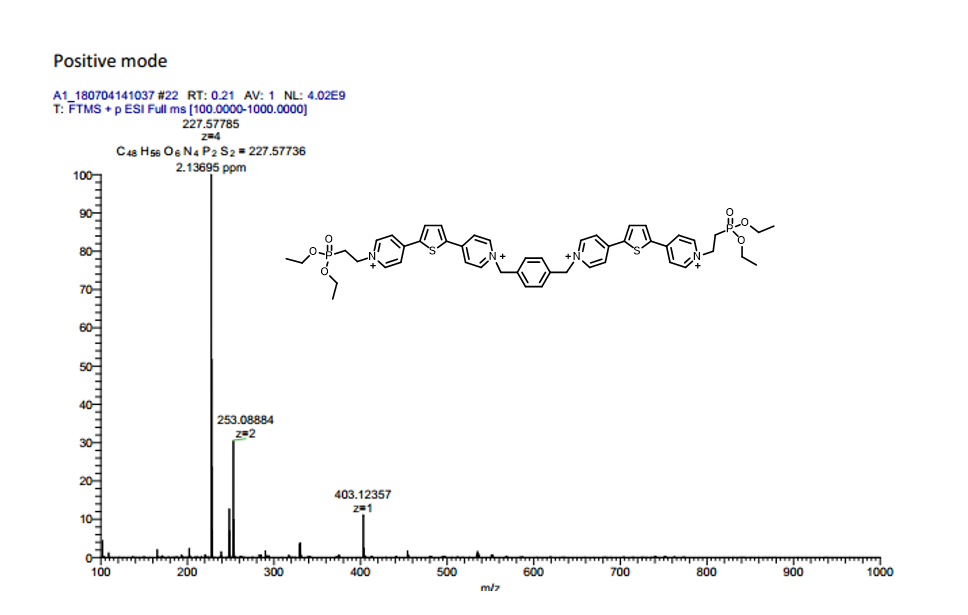
**

**Figure S5** **Characterization of the Compound 7.**  HRMS of **Compound 7**


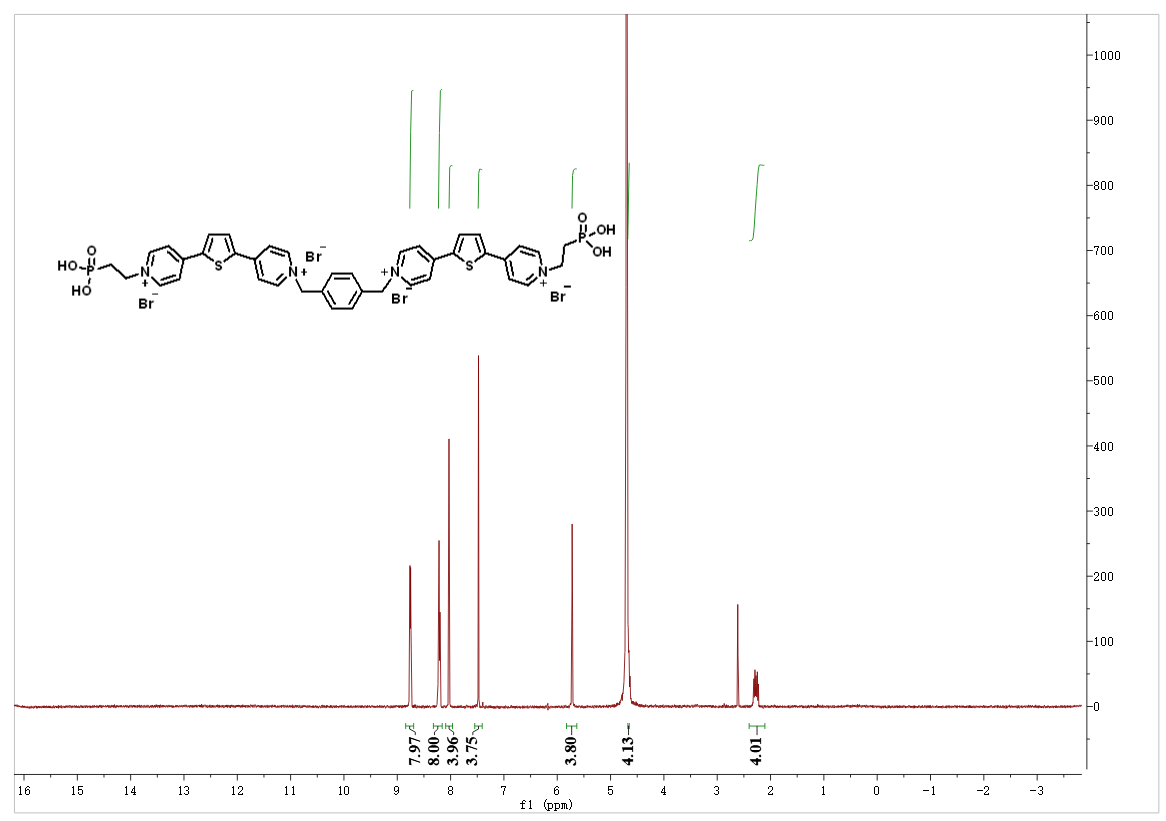


**Figure S6** **Characterization of the TGP.** ^1^H NMR of **TGP**

**
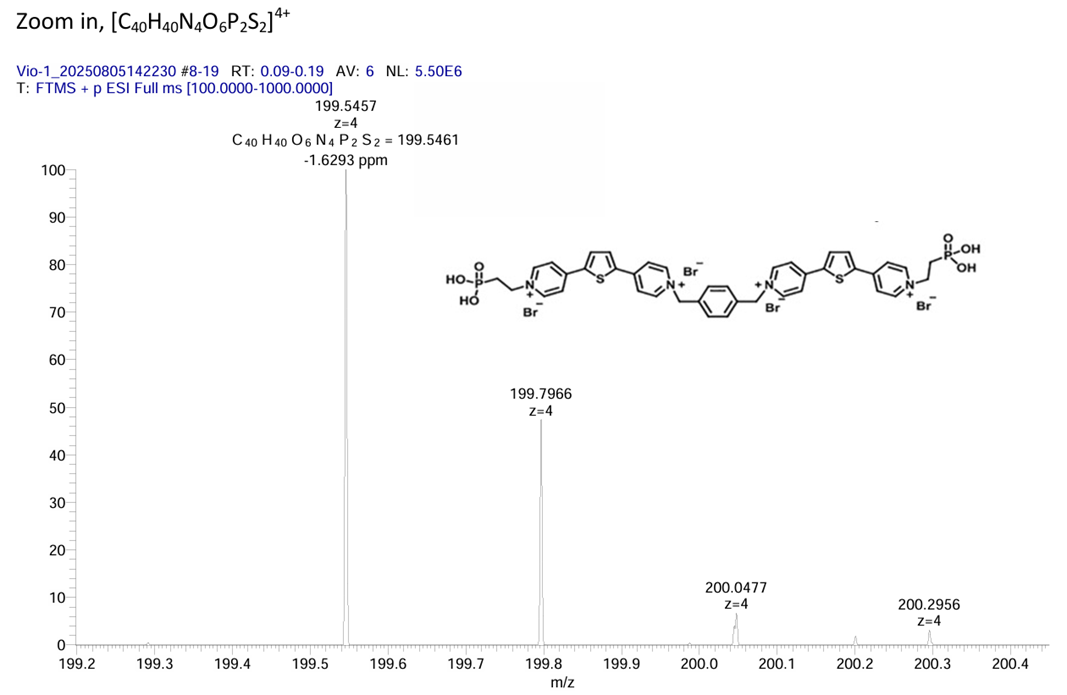
**

**Figure S7** **Characterization of the TGP.**  HRMS of **TGP**

**
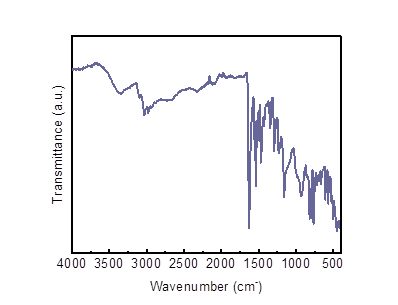
**

**Figure S8** **Characterization of the TGP.**  FTIR of **TGP**

**Fabrication of TT-EESD**

**Figure S9** illustrates the fabrication process of TT-EESD. Initially, TiO_2_ nanopastes were put onto FTO conductive glass using a blade-coating technique. The coated FTO glass was then sintered at 500°C for 30 minutes to achieve a uniform film. Following this, the FTO/TiO_2_ glass was submerged in a 0.1 mM **TGP** aqueous solution at room temperature for a duration of 24 hours. Subsequently, it underwent a thorough rinse with absolute ethyl alcohol for three times and was dried on a thermal plate at 100 °C for 10 minutes to remove any residual solvent. Next, a metal Zn frame was securely attached around the perimeter of the FTO glass using double-sided tape. Upon drop-casting 1 M Zn(OTF)_2_ liquid electrolyte onto the central area, a piece of non-conductive glass was positioned over it. Finally, the assembled TT-EESD was sealed with UV-curable glue to prevent its leakage.


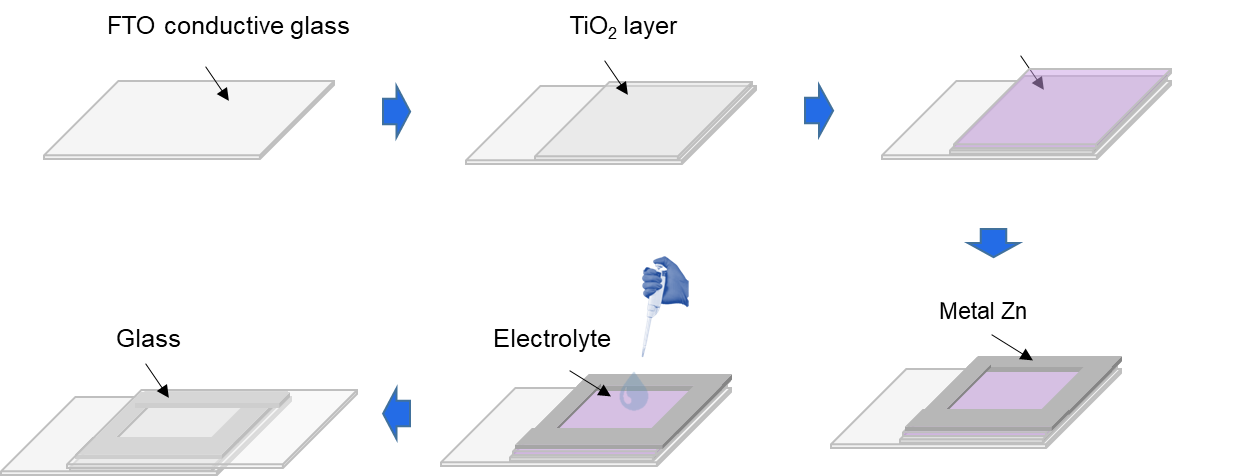


**Figure S9** **Schematics of the fabrication process of TT-EESD.** The TT-EESDs are composed of FTO glass, a TiO₂ film, **TGP** molecules, a Zn framework, and an electrolyte.

**Characterization**

Scanning electron microscope (SEM, MERLIN Compact, ZEISS, Germany) was used to characterize the microstructures of TiO_2_ film. Transmission electron microscope (TEM, F30, FEI, USA）equipped with an energy dispersive spectrometer was utilized to measure the element composition of the samples. The chemical states were tested using X-ray photoelectron spectroscopy (XPS) (PerkinElmer Corp., USA) equipped with an AlKα x-ray (1486.6 eV) source. Transmittance measurement was performed by using a UV-visible-near infrared spectrophotometer (Lambda 650S, PerkinElmer, USA) from 400 to 800 nm. The crystallographic structures of the TiO_2_ films were examined by a Rigaku Smartlab X-ray diffractometer (XRD, Rigaku, Japan) with Cu Kα radiation ranging from 20° to 80°. All the electrochemical performances were evaluated by an electrochemical workstation (CHI 760E, Chenhua, USA). The electrochemical performances were measured in a two-electrode system, and the reference electrode and counter electrode were connected. The coloration efficiency was calculated using the equation (1):

CE =ΔOD/ΔQ =log (T_b_/T_c_) /ΔQ (1)

In which ΔOD is the optical density, T_b_ and T_c_ are the transmittance at bleached and colored state. ∆Q is charging density. The response time was defined the time required to reach 80% of the total optical modulation range.

***Supplied Note 2***

**The electrochemical process during the operation of TT-EESDs**

In our electrochromic energy storage device, a hybrid electrode composed of a nanoporous TiO_2_ film chemically bonded with a viologen derivative (**TGP**) functions as the cathode, while a zinc (Zn) foil serves as the anode. During discharge, Zn is oxidized at the anode:

Zn→Zn^2+^+2e^-^

The released electrons flow through the external circuit to the TiO₂–TGP electrode, where they participate in two simultaneous electrochemical processes:

1. Electrochromic color switching:

The viologen species (TGP^2(2+)^) are electrochemically reduced to their radical cation form TGP^2(+^**^·^**^)^, accompanied by an intense color change (from transparent to magenta):

TGP^2(2+)^+2e^-^−→TGP^2(+^**^·^**^)^

To maintain charge neutrality, anions from the electrolyte, OTF^-^, are doped into the electrode structure during this redox process.

1. Energy storage via TiO₂ intercalation:

Simultaneously, Zn^2+^ ions from the electrolyte are intercalated into the TiO₂ matrix:

TiO_2_+xZn^2+^+2xe^-^→Zn_x_TiO_2_

During charging, these reactions are reversed: Zn^2+^ ions are deintercalated from TiO₂ and reduced at the anode to reform metallic Zn; TGP^2(+^**^·^**^)^ is oxidized back to the colorless TGP^2(2+)^ form:

TGP^2(+^**^·^**^)^→TGP^2(2+)^+e^-^

***Supplied Figures and Captions***

**Figure S10** **FTIR spectra of TiO_2_, TiO_2_/TGP.** A distinct peak observed at 1083 cm⁻¹, which corresponds to Ti–O–P bonds and confirms the successful anchoring of TGP molecules onto the TiO₂ surface via chemical bonding.


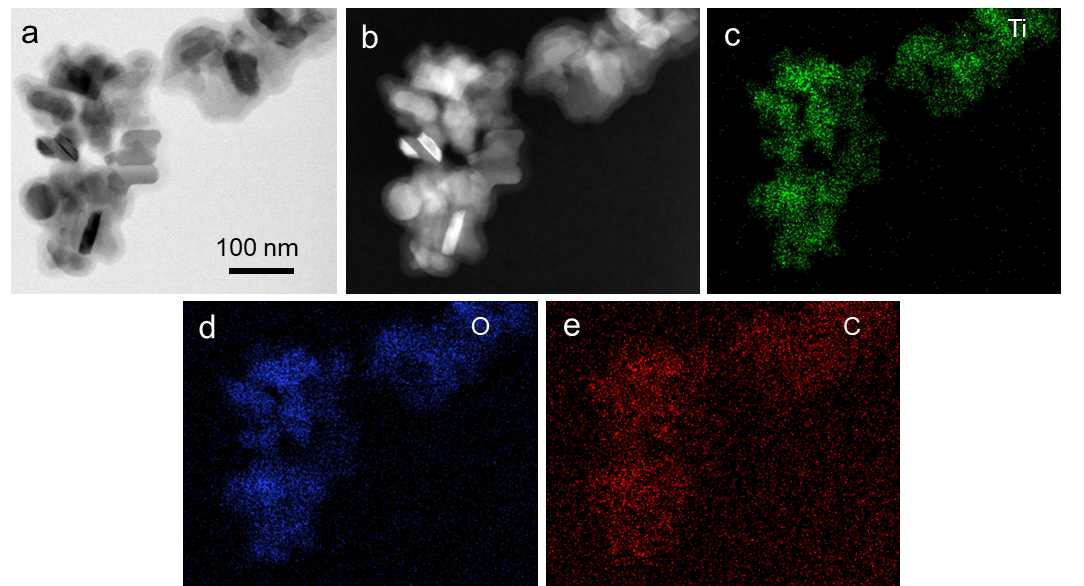


**Figure S11** **EDS analysis of TiO_2_ nanoparticles after adsorbing TGP molecules.** (a) TEM images; (b) STEM images; (c–e) Elemental distribution of Ti (c), O (d) and C (e). The distribution area of C is slightly larger than that of elements Ti and O, indicating that **TGP** molecules are adsorbed onto the surface of TiO_2_ nanoparticles.


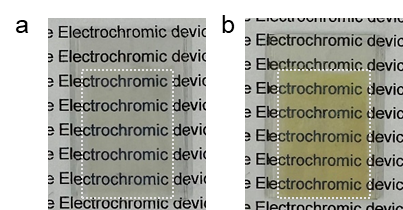


**Figure S12** **Optical characterization of TiO_2_ and TiO_2_/TGP films.** (a) TiO_2_, (b) **TGP** film. The observed color change from white to yellow also confirms the adsorbing of **TGP** molecules onto TiO_2_ film.


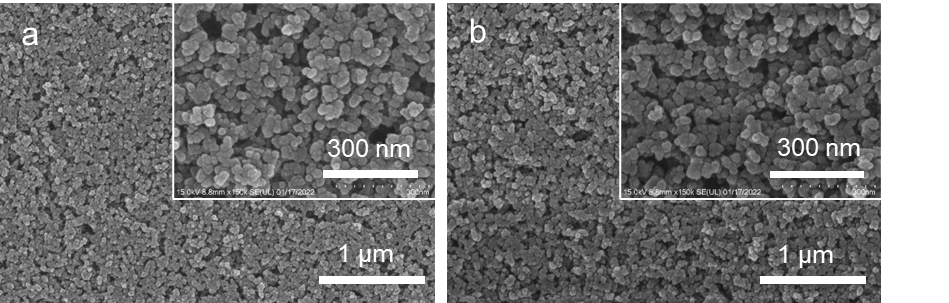


**Figure S13** **SEM Characterization of TiO_2_ and TiO_2_/TGP film.** (a) TiO_2_, (b) **TGP** film. No obvious change was observed after adsorbing **TGP**, confirming a thin and uniform **TGP** layer was coated onto TiO_2_ film.


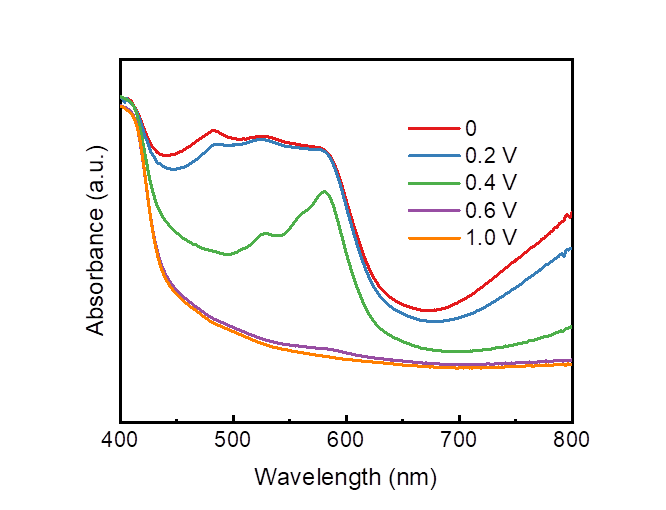


**Figure S14** **Absorbance spectrum of a TT-EESD.** As the inputted voltage decrease, the TT-EESD exhibits a more pronounced absorption peak at 580 nm, corresponded to the decreased transmittance and the coloring process.


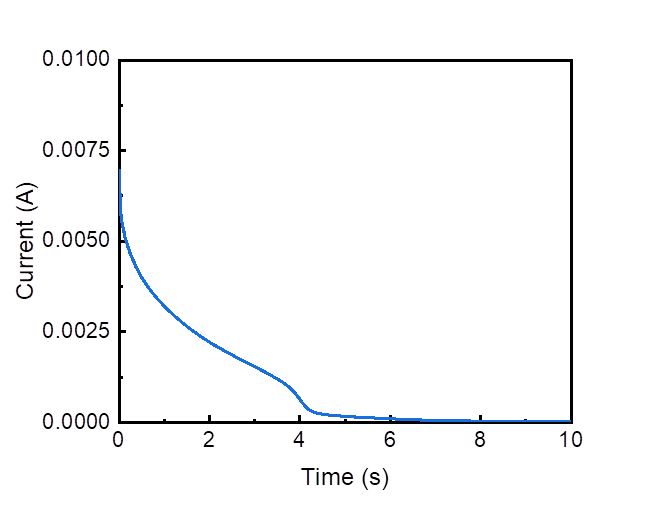


**Figure S15** **Chronoamperometry (CA) curve of a TT-EESD.**  The CA curves were recorded in a voltage window from 0 V to 1 V. The injected charges can be calculated by the integral of CA curves, which is an important parameter for calculating coloration efficiency.


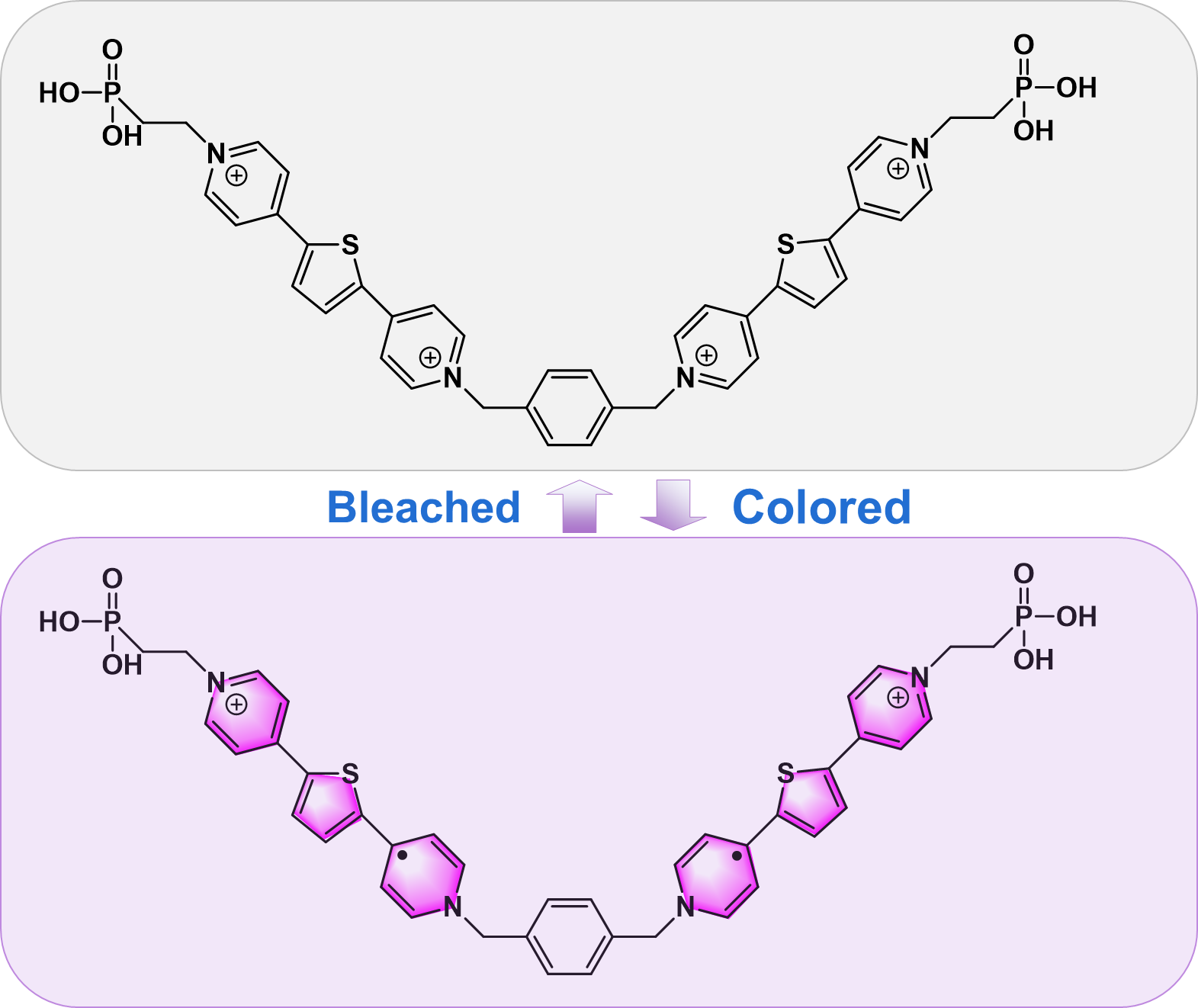


**Figure S16** **Electrochromic mechanism of TGP**. Electrochromism is caused by the reversible transition between the colorless dicationic state (**TGP**^2(++)^) and the magenta-colored radical cation state (**TGP**^2(+•)^).


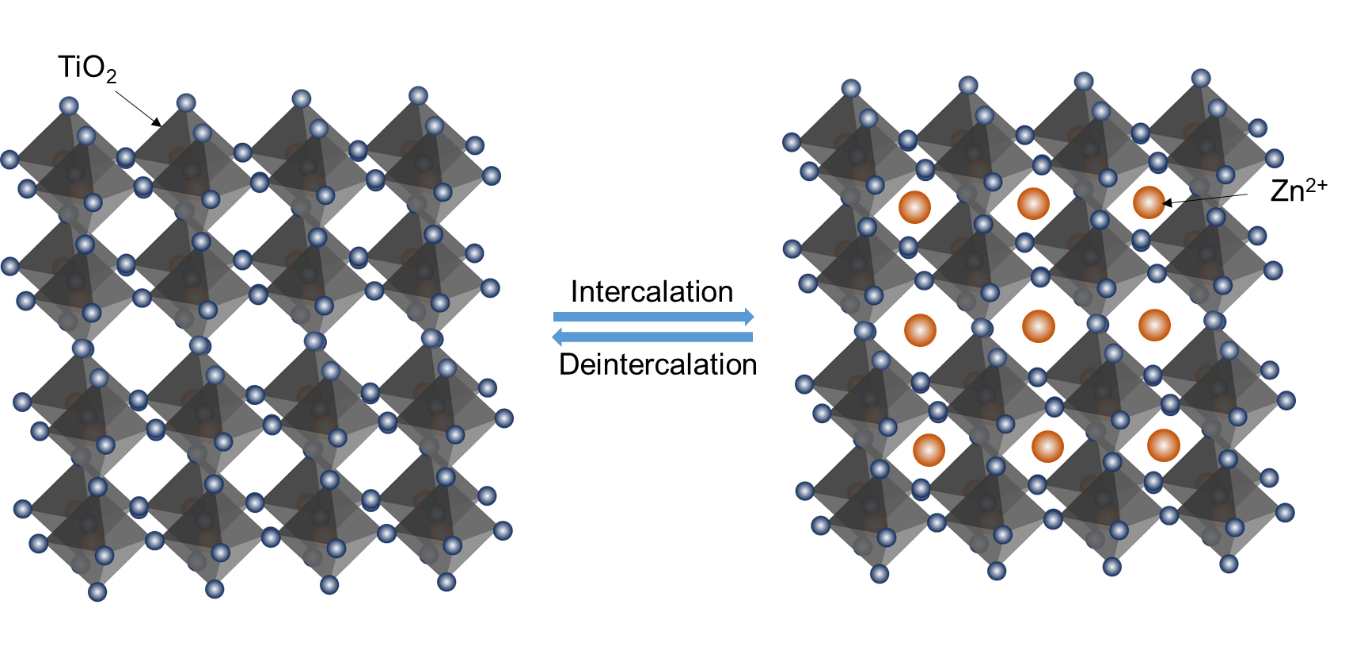


**Figure S17** **Energy storage mechanism of TGP**. Schematic illustration of intercalation/deintercalation of Zn^2+^ in TiO_2_.


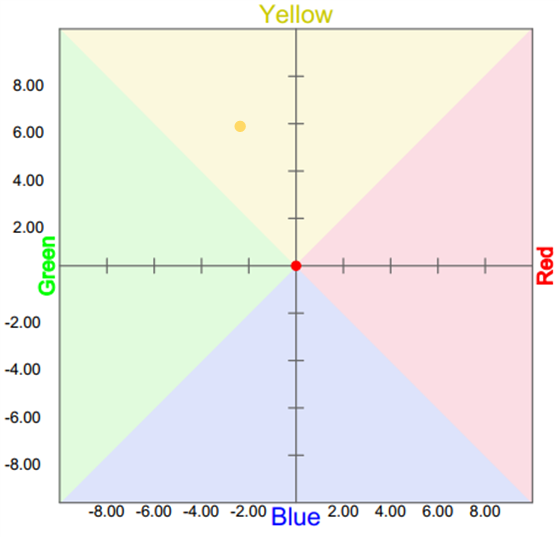


**Figure S18** **Quantitative color characterization of a TT-EESD**. The plot of b* *vs.* a* at colored (left) and bleached state (right). From bleached state to colored state, the point shift from the yellow region to red region, which corresponds with the color changing from light yellow to magenta.

**Figure S19** **Electrochromic stability of TT-EESD.** The transmittance of TT-EESDs after 3000 color-bleach cycles in a voltage range of 0 V to 1 V show negligible deviation from the initial state (as shown in **Figure 2a**), demonstrating the excellent stability of the TT-EESDs.


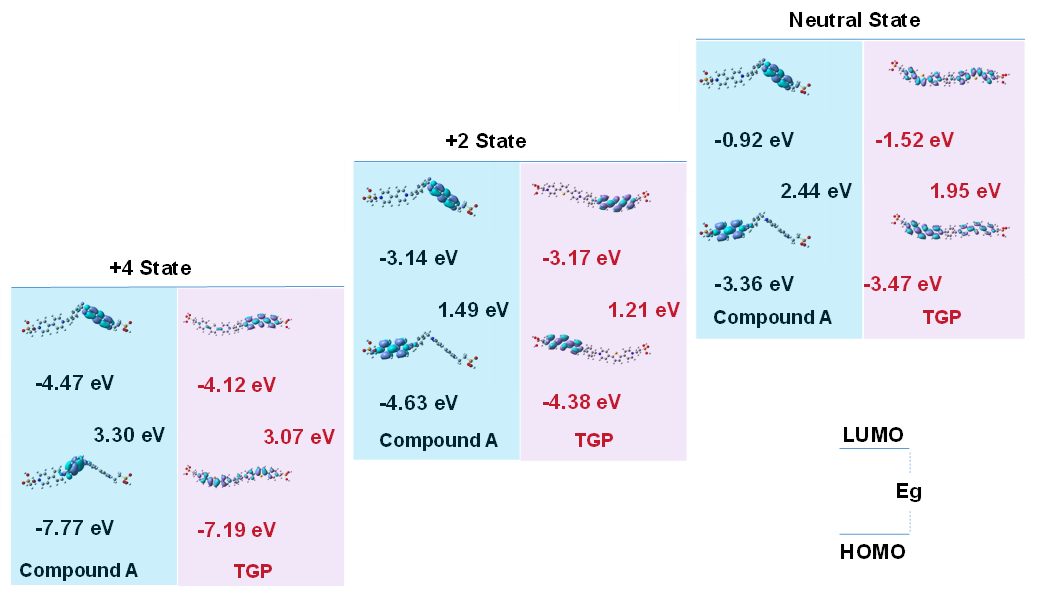


**Figure S20** **Calculation of the band gaps.** The HOMO, LUMO, and *E_g_* values of **TGP** and **Compound A** in different valence states are compared. Regardless of the chemical state, **TGP** exhibits a smaller *E_g_* ​ than that of Compound **A**, which benefits redox reversibility.


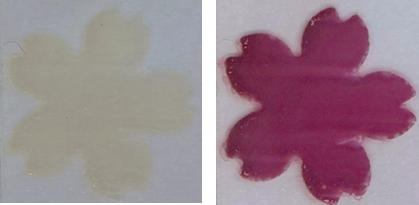


**Figure S21** **Patterned TT-EESD.** Flower-patterned TT-EESDs at bleached (left) and colored (right) state.


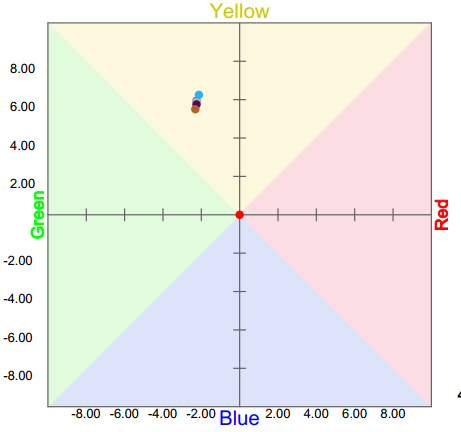

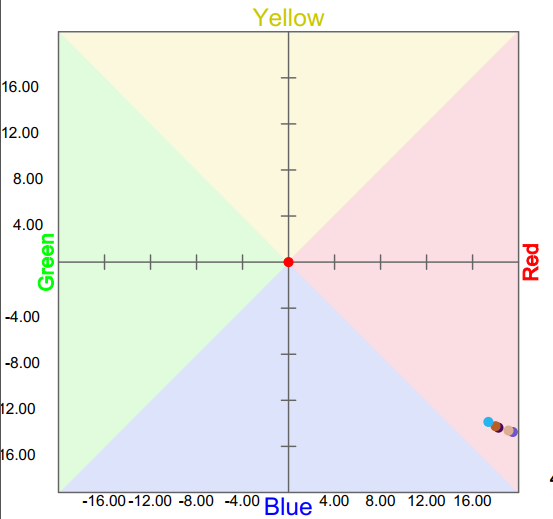


**Figure S22** **Uniformity testing of large-area TT-EESD**. The plot of b* *vs.* a* in the bleached (left) and colored (right) states of five different regions shown in Figure S17.


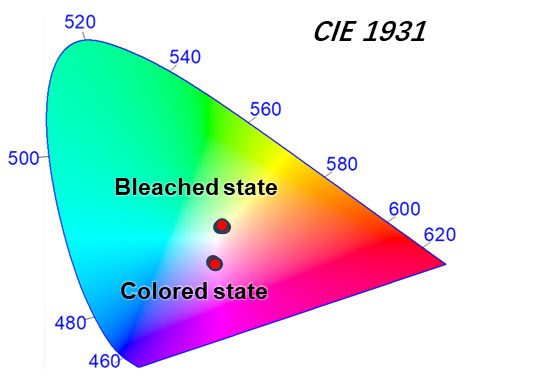


**Figure S23** **Uniformity testing of large-area TT-EESD**. Peak*x*y* color space 481 (CIE 1931) results of TT-EESDs at colored and bleached states in five different regions.

**Figure S24** **Working mechanism of TT-ESSDs with Zn anode**. (a–b) Comparison of working principles of conventional ECD (a) and TT-EESD (b) with Zn anode. For conventional ECDs, during both the coloring or bleaching processes, external power sources are required. However, in TT-EESDs, the coloring process can be achieved through the discharging method. When the two electrodes are short-circuited, the TT-EESDs can be rapidly colored.

**Figure S25** **The energy consumption of a TT-EESD during the coloring process.** In the Zn-anode TT-EESD, the coloring process was realized by short-circuiting the two electrodes, indicating that the energy consumed, 11.5 mWh/cm^2^, during a single coloring cycle can be effectively recovered.

**Figure S26** **Calculation of ionic diffusion coefficient.** (a) Ionic diffusion coefficient of TiO_2_/**TGP**. (b) Plot of log *i* vs. log *v*.


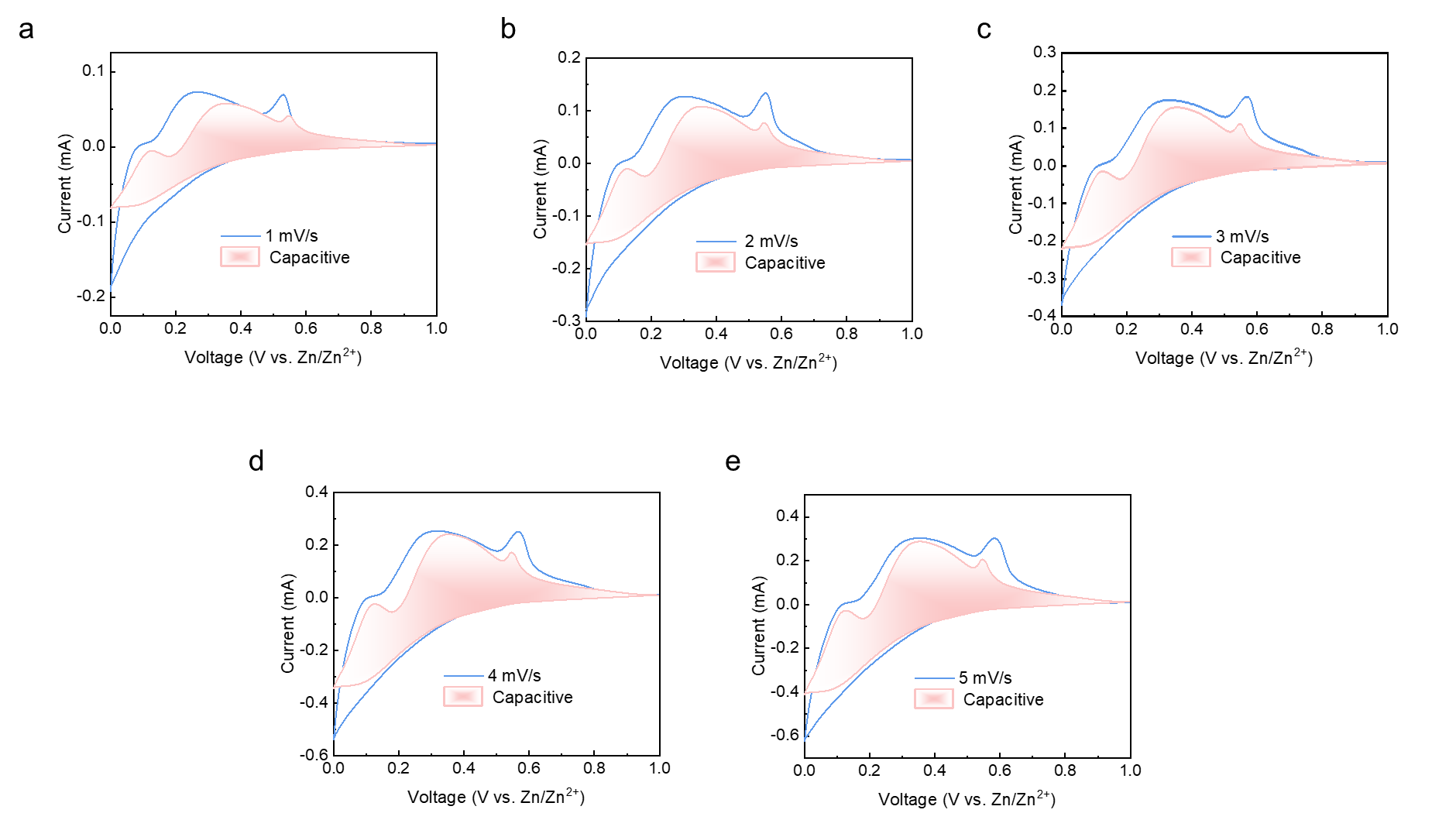


**Figure S27** **Calculation of the capacitive contribution.** The red regions show the capacitive contribution at different scan rates: (a) 1 mV/s; (b) 2 mV/s; (c) 3 mV/s; (d) 4 mV/s; (e) 5 mV/s. The current of CV curves at each potential was assumed to have two contributions:i(V)=k_1_v+k_2_v^1/2^ where k_1_v represents capacitive (surface-controlled) current and k_2_v^1/2^ represents diffusion-controlled current. For each potential, a plot of i(V)/ v^1/2^ *vs.* v^1/2^ was fitted linearly to extract k_1_ and k_2_. The capacitive current (k_1_v) was integrated over the entire potential range and expressed as a percentage of the total CV area. Then, the capacitive contribution at different scan rates was plotted to obtain the updated contribution ratio curves. These corrections ensure both the accuracy of the capacitive contribution analysis and consistency between figures and captions.


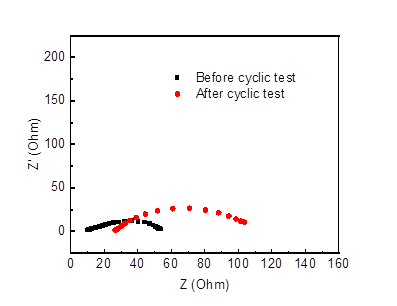


**Figure S28** **Electrochemical impedance spectroscopy (EIS)**. The Nyquist plots show a slight increase in the semicircle diameter after cycling, indicating a higher charge transfer resistance, which is also corresponded with the increase of switching time after cycling tests. Notably, the switching time (t_b_/t_c_) increased to 4.5 s/5.5 s after 3000 times cycles.

**Figure S29** **The discharge capacity at different current densities.** With the increase in current density, the discharge capacity gradually decreased, suggesting the excellent rate performance.

**Figure S30** **The Ragone plot of TT-EESDs.** The TT-EESDs exhibited excellent energy storage performance. For example, it shows a superior energy density of 31.11 mWh/m^2^ at a power density of ~1000 mW/m^2^.


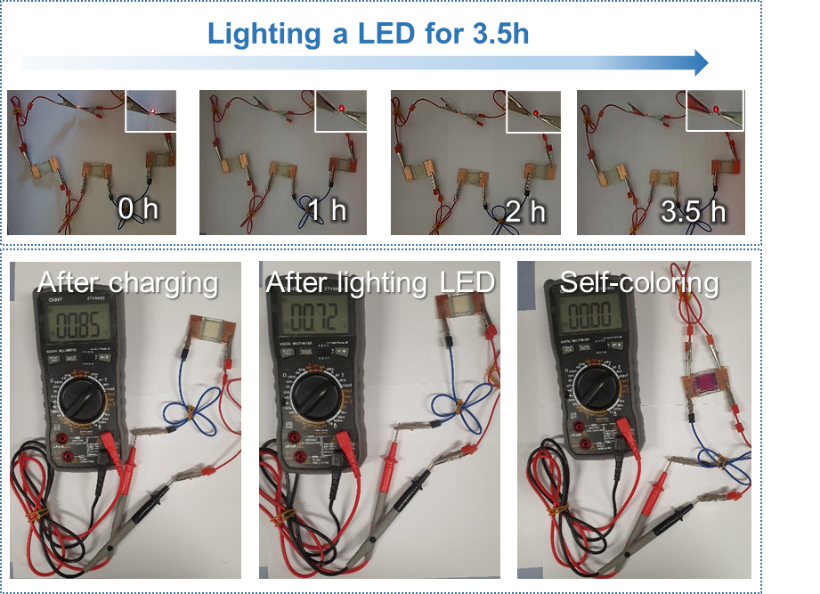


**Figure S31** **The practical application of prototype devices.** After powering an LED for 3.5 h, the voltage of TT-EESDs only exhibited a mild decrease.

**Figure S32** **Energy storage stability of TT-EESD.** The discharge curve of TT-EESDs after 3000 charging-discharging cycles within a voltage window of 0 V to 1 V, which demonstrates the excellent stability of the TT-EESDs.

***Supplied Tables and Captions***

**Table S1** A Comparison of this work with previous reports

| **Materials** | **Synthesizing**  **method** | **CE**  **(cm^2^/C)** | **Capacity**  **(mAh/m^2^)** | **Optical modulation**  **(%)** | **@λ (nm)** | **t_c_/t_b_ (s)** | **Ref.** |
| --- | --- | --- | --- | --- | --- | --- | --- |
| TiO_2_ | Solvothermal | 84.96 | 2.1 | NA | 600 | 20/12 | 1 |
| Ni(OH)2/Ni(Co)_2_ | Electrodeposition | 59.89 | 71.5 | 55.69 | 550 | NA | 2 |
| Metal-organic coordination polymer | Organic chemical synthesis | 66 | 27.06 | 54 | 517 | 5.8/4.8 | 3 |
| polyaniline | Electrodeposition | 112.8 | 20 | 75.7 | 666 | 6.9/6.3 | 4 |
| PANI/WO_3_ | Electrodeposition | 153.77 | 11.45 | 30 | 633 | NA | 5 |
| Prussian white@MnO_2_ | Electrodeposition/  Sputtering | 77.6 | 71.25 | 35 | 510 | 15/11 | 6 |
| Prussian blue | Hydrothermal | 76.8 | 78.9 | 84.9 | 633 | 4.1/4.6 | 7 |
| Prussian blue/organic dye | Hydrothermal | 265 | 84 | 81.4 | 670 | NA | 8 |
| Prussian blue | Chemical Bath Deposition | 117.2 | 45.8 | 68.3 | 700 | 4.7/7.5 | 9 |
| Ethyl viologen | Commerical | 106 | 25 | 40.3 | 605 | NA | 10 |
| WO_3_ | Hydrothermal | NA | 50 | 50 | 700 | 18.7/11.4 | 11 |
| TiO_2_ | Sonochemical synthesis | 112 | 40.75 | 95.5 | 633 | 15.5/3.4 | 12 |
| Electron donor–acceptor polymer | Electropolymerization | 79.8 | 110(mAh/g) | 44.2 | 550 | NA | 13 |
| WO_3_·0.5H_2_O | Photodeposition | 61.9 | 209.8 | 69 | 633 | 7.0/3.7 | 14 |
| WO_3_ | Electrodeposition | NA | 126.3 | 88 | 633 | 5.7/10.3 | 15 |
| Nb_18_W1_6_O_93_ | spin-coating presursor | 45.28 | 106.7 | 90 | 633 | 10.8/25.1 | 16 |
| Li_4_Ti_5_O_12_ | spin-coating presursor | 38.62 | 151.94 | 76 | 633 | 7.7/4.4 | 17 |
| WO_3_ | thermal evaporation | 80.2 | 18.9 | 44.1 | 633 | 1.7/1.0 | 18 |
| rGO/W_18_O_49_-polyaniline | Solvothermal-electrodeposition | 76.37 | 54.4 | ~20 | 632 | 18/13 | 19 |
| TiO_2_/**TGP** | Chemical bonding | 512.93 | 62.2 | 53 | 580 | 4.0/3.6 | This work |

**Table S2** CIE parameters of the TT-EESDs at colored and bleached state

| State | Colored | Bleached |
| --- | --- | --- |
| L* | 46.51 | 72.65 |
| a* | 19.79 | -2.43 |
| b* | -14.46 | 6.45 |

**Table S3** Chemical formulas and optimized structures of Compound **A** and **TGP**.

**Table S4** CIE parameters of the TT-EESDs at colored and bleached states in Figure S19

| Region | Colored  (L*, a, b) | Bleached  (L*, a, b) |
| --- | --- | --- |
| 1 | (48.54, 19.33, -14.28) | (72.13, -2.43, 6.45) |
| 2 | (48.86, 18.92, -14.15) | (72.51, -2.46, 6.38) |
| 3 | (49.45, 18.12, -13.83) | (72.11, -2.41, 6.24) |
| 4 | (49.69, 17.81, -13.69) | (72.03, -2.52, 6.03) |
| 5 | (48.54, 19.33, -13.40) | (71.70, -2.30, 6.72) |

**Table S5** The detailed discharge capacity at different scan rates.

| Current density (mA/cm^2^) | 0.2 | 0.4 | 0.6 | 0.8 | 1.0 | 1.2 |
| --- | --- | --- | --- | --- | --- | --- |
| Discharge capacity (mAh/cm^2^) | 62.22 | 53.38 | 46.7 | 37.8 | 30.8 | 26.69 |

**Table S6** The detailed energy densities at different power densities.

| Power density (mW/m^2^) | 999.96 | 2001.75 | 3002.14 | 4002.35 | 5040.00 | 6005.25 |
| --- | --- | --- | --- | --- | --- | --- |
| Energy density (mWh/m^2^) | 62.22 | 53.38 | 46.7 | 37.8 | 30.8 | 26.69 |

**Reference**

1. Xing C, Yang L, He R, et al. Brookite TiO2 Nanorods as Promising Electrochromic and Energy Storage Materials for Smart Windows. *Small*. 2023/12/01 2023;19(49):2303639. doi:<https://doi.org/10.1002/smll.202303639>

2. Xuan X, Qian M, Pan L, et al. A NiCo bimetallic hydroxide electrode-based flexible Ni//Zn battery with smart electrochromic function for visually monitoring battery residual electricity. *Science China Materials*. 2023/02/01 2023;66(2):567-576. doi:10.1007/s40843-022-2174-y

3. Cong B, Xie Y, Wu Y, et al. Metal-organic coordination polymer bearing dual-redox centra enables high-performance electrochromic supercapacitor. *Chemical Engineering Journal*. 2023/10/15/ 2023;474:145528. doi:<https://doi.org/10.1016/j.cej.2023.145528>

4. Wang Y, Zhong X, Liu X, et al. A fast self-charging and temperature adaptive electrochromic energy storage device. 10.1039/D1TA10726G. *Journal of Materials Chemistry A*. 2022;10(8):3944-3952. doi:10.1039/D1TA10726G

5. Zhang H, Tian Y, Wang S, et al. Robust Cu-Au alloy nanowires flexible transparent electrode for asymmetric electrochromic energy storage device. *Chemical Engineering Journal*. 2021/12/15/ 2021;426:131438. doi:<https://doi.org/10.1016/j.cej.2021.131438>

6. Ding Y, Wang M, Mei Z, Diao X. Novel Prussian White@MnO2-Based Inorganic Electrochromic Energy Storage Devices with Integrated Flexibility, Multicolor, and Long Life. *ACS Applied Materials & Interfaces*. 2022/11/02 2022;14(43):48833-48843. doi:10.1021/acsami.2c12484

7. Wang B, Cui M, Gao Y, et al. A Long-Life Battery-Type Electrochromic Window with Remarkable Energy Storage Ability. *Solar RRL*. 2020/03/01 2020;4(3):1900425. doi:<https://doi.org/10.1002/solr.201900425>

8. Xu M, Wu T, Yin K, et al. Organic Dye Molecule Intercalated Prussian Blue for Simultaneously Enhancing Coloration Efficiency and Energy Storage Capacity in Electrochromic Battery. *Small Methods*. 2024/10/14 2024;n/a(n/a):2401188. doi:<https://doi.org/10.1002/smtd.202401188>

9. Ding Y, Sun H, Li Z, et al. Galvanic-driven deposition of large-area Prussian blue films for flexible battery-type electrochromic devices. 10.1039/D2TA08023K. *Journal of Materials Chemistry A*. 2023;11(6):2868-2875. doi:10.1039/D2TA08023K

10. Zhang H, Sun F, Feng J, et al. A stable, self-regulating, flexible, ITO-free electrochromic smart window for energy-efficient buildings. *Cell Reports Physical Science*. 2022;3(12)doi:10.1016/j.xcrp.2022.101193

11. Roy R, R G, Basith A, Banerjee R, Singh AK. Self-rechargeable aqueous Zn2+/K+ electrochromic energy storage device via scalable spray-coating integrated with marangoni flow. *Energy Storage Materials*. 2024/08/01/ 2024;71:103680. doi:<https://doi.org/10.1016/j.ensm.2024.103680>

12. Zhang S, Cao S, Zhang T, Lee JY. Plasmonic Oxygen-Deficient TiO2-x Nanocrystals for Dual-Band Electrochromic Smart Windows with Efficient Energy Recycling. *Advanced Materials*. 2020/10/01 2020;32(43):2004686. doi:<https://doi.org/10.1002/adma.202004686>

13. Yun TG, Lee J, Kim HS, et al. A π-Bridge Spacer Embedded Electron Donor–Acceptor Polymer for Flexible Electrochromic Zn-Ion Batteries. *Advanced Materials*. 2023/08/01 2023;35(31):2301141. doi:<https://doi.org/10.1002/adma.202301141>

14. Zhuang D, Zhang Z, Weng J, Wang J, Zhang H, Cheng W. Amorphous Hydrated Tungsten Oxides with Enhanced Pseudocapacitive Contribution for Aqueous Zinc-Ion Electrochromic Energy Storage. *Advanced Energy Materials*. 2024/10/01 2024;14(40):2402603. doi:<https://doi.org/10.1002/aenm.202402603>

15. Li H, Firby CJ, Elezzabi AY. Rechargeable Aqueous Hybrid Zn2+/Al3+ Electrochromic Batteries. *Joule*. 2019/09/18/ 2019;3(9):2268-2278. doi:<https://doi.org/10.1016/j.joule.2019.06.021>

16. Wu C, Shi H, Zhao L, et al. High-Performance Aqueous Zn2+/Al3+ Electrochromic Batteries based on Niobium Tungsten Oxides. *Advanced Functional Materials*. 2023/05/01 2023;33(20):2214886. doi:<https://doi.org/10.1002/adfm.202214886>

17. Wu Z, Lian Z, Yan S, et al. Extraordinarily Stable Aqueous Electrochromic Battery Based on Li4Ti5O12 and Hybrid Al3+/Zn2+ Electrolyte. *ACS Nano*. 2022/08/23 2022;16(8):13199-13210. doi:10.1021/acsnano.2c06479

18. Shen L, Du L, Tan S, Zang Z, Zhao C, Mai W. Flexible electrochromic supercapacitor hybrid electrodes based on tungsten oxide films and silver nanowires. *Chem Commun (Camb)*. May 7 2016;52(37):6296-9. doi:10.1039/c6cc01139j

19. Li Y, Yan L, Zhang L, Song X, Dai C. Design of electrochromic supercapacitor based on rGO–W18O49 nanowires/polyaniline. *Journal of Materials Science: Materials in Electronics*. 2021/07/01 2021;32(14):19179-19190. doi:10.1007/s10854-021-06439-8
